# Late plasma exosome microRNA-21-5p depicts magnitude of reverse ventricular remodeling after early surgical repair of primary mitral valve regurgitation

**DOI:** 10.3389/fcvm.2022.943068

**Published:** 2022-07-29

**Authors:** Fausto Pizzino, Giulia Furini, Valentina Casieri, Massimiliano Mariani, Giacomo Bianchi, Simona Storti, Dante Chiappino, Stefano Maffei, Marco Solinas, Giovanni Donato Aquaro, Vincenzo Lionetti

**Affiliations:** ^1^Unit of Translational Critical Care Medicine, Scuola Superiore Sant’Anna, Pisa, Italy; ^2^Fondazione Toscana Gabriele Monasterio, Pisa, Italy

**Keywords:** exosomes, reverse remodeling, mitral valve (MV) repair, heart surgery, postoperative, miR-21-5p

## Abstract

**Introduction:**

Primary mitral valve regurgitation (MR) results from degeneration of mitral valve apparatus. Mechanisms leading to incomplete postoperative left ventricular (LV) reverse remodeling (Rev–Rem) despite timely and successful surgical mitral valve repair (MVR) remain unknown. Plasma exosomes (pEXOs) are smallest nanovesicles exerting early postoperative cardioprotection. We hypothesized that late plasma exosomal microRNAs (miRs) contribute to Rev–Rem during the late postoperative period.

**Methods:**

Primary MR patients (*n* = 19; age, 45–71 years) underwent cardiac magnetic resonance imaging and blood sampling before (T0) and 6 months after (T1) MVR. The postoperative LV Rev–Rem was assessed in terms of a decrease in LV end-diastolic volume and patients were stratified into high (HiR-REM) and low (LoR-REM) LV Rev–Rem subgroups. Isolated pEXOs were quantified by nanoparticle tracking analysis. Exosomal microRNA (miR)-1, –21–5p, –133a, and –208a levels were measured by RT-qPCR. Anti-hypertrophic effects of pEXOs were tested in HL-1 cardiomyocytes cultured with angiotensin II (AngII, 1 μM for 48 h).

**Results:**

Surgery zeroed out volume regurgitation in all patients. Although preoperative pEXOs were similar in both groups, pEXO levels increased after MVR in HiR-REM patients (+0.75-fold, *p* = 0.016), who showed lower cardiac mass index (–11%, *p* = 0.032). Postoperative exosomal miR-21-5p values of HiR-REM patients were higher than other groups (*p* < 0.05). *In vitro*, T1-pEXOs isolated from LoR-REM patients boosted the AngII-induced cardiomyocyte hypertrophy, but not postoperative exosomes of HiR-REM. This adaptive effect was counteracted by miR-21-5p inhibition.

**Summary/Conclusion:**

High levels of miR-21-5p-enriched pEXOs during the late postoperative period depict higher LV Rev–Rem after MVR. miR-21-5p-enriched pEXOs may be helpful to predict and to treat incomplete LV Rev–Rem after successful early surgical MVR.

## Introduction

Mitral valve regurgitation (MR) is one of the most common cardiac valve diseases leading to blood regurgitation into the left atrium (LA) and to left ventricular (LV) dilatation, also defined as “LV adverse remodeling,” following increased preload up to the onset of heart failure (HF) (1). Primary MR is associated with degeneration of the valve apparatus or with annular dilatation. Even though patients with a severe primary MR remain asymptomatic for a remarkably long period of time with preserved LV function (2–5), an increased risk for cardiovascular morbidity and mortality is described (2, 4, 6). Therefore, early surgical mitral valve repair (MVR) is strongly recommended in experienced surgical center by current guidelines (7) due to the lack of effective pharmacological treatments. Indeed, long-term postoperative survival is worse if surgery is performed after patients become symptomatic and the mitral valve is not successfully repairable.

Most patients with severe primary MR and LV dysfunction undergoing timely MVR (7–9) are at minimal risk of MR recurrence and show progressive reduction of LV volumes defined as “LV reverse remodeling (Rev–Rem)” (10–12). However, a non-negligible number of patients may experience worsened clinical outcome during late postoperative period due to a partial LV Rev–Rem (13–15) after timely and technically successful MVR (16, 17). Early recognition and treatment of these patients remains a desirable goal in hospital setting (4, 18, 19). Indeed, personalization of early-to-late postoperative follow-up and timely escalation of therapy for patients at higher risk for incomplete LV Rev–Rem is expected even when patients with severe MR, preserved LV function, and normal exercise capacity undergo early surgical treatment (7, 9).

Preoperative echocardiography is now commonly used as a method for screening postoperative outcome in terms of long-term survival (20–23), MR recurrence (24), Rev–Rem (12, 25–27) and functional preservation of the left ventricle (13, 14, 28–30). However, the early predictive value of echocardiography is affected by several limitations (31, 32). Conversely, cardiac magnetic resonance imaging (CMRI) is the gold-standard technique in the evaluation of cardiac mass, fibrosis, volumes, dimensions and function (33, 34) and is highly reliable in the characterization of cardiac tissues and in the evaluation of LV remodeling. Yet, its role in depicting the pathogenic events that occur at the molecular level to predict early-to-late LV Rev–Rem after early rescue mitral valve surgery is still undefined (35). Moreover, the perioperative use of CMRI is limited by the low availability of the method, and by the high cost. Despite the availability of present indications for early rescue mitral valve surgery (36) and current non-invasive diagnostic technologies, the quality of surgical outcomes in patients with primary MR remains heterogeneous, and the traditional markers (symptoms, LV phenotype) are poor outcome markers. Therefore, additional reliable and more sensitive indicators of reverse ventricular remodeling during medical management are of paramount importance to start treating adverse myocardial remodeling in the postoperative period even in the absence of cardiac dysfunction and symptoms.

Plasma exosomes (pEXOs) are smallest membrane bound extracellular vesicles (40–150 nm in diameter) released by different cells, which may play a key role in intercellular communication by regulating the magnitude of postoperative LV Rev–Rem. It is conceivable that combining analysis of pEXOs with CMRI parameters may enable more precise and informative assessment of late LV Rev–Rem. Indeed, the previous studies have demonstrated the role of pEXOs in mediating cardioprotection through anti-inflammatory and anti-apoptotic pathways (37, 38) regardless of the type of cardiac surgery (39). Although the investigation on their role in late LV Rev–Rem after surgery is at its infancy, changes of pEXOs profile have been already suggested as non-invasive early indicators of cardiac function in both critically ill (40) and surgical patients (41, 42), even after the heart transplantation (43).

Exosomal microRNA (miR), small non-coding RNA molecule (∼20 nucleotides) regulating gene expression, is effective mediator of the adaptive paracrine responses in cardiomyocytes exposed to different stressors (38, 44). The distinctive microRNAs expression patterns are associated with MR (45) and exosomes are the source of choice for microRNAs in biomarker studies (46, 47). In fact, the exosomal fraction of microRNAs was able to predict the risk of adverse cardiac events in patients with stable coronary artery diseases (48) and was correlated with postoperative cardiac troponin levels in patients subjected to coronary artery–bypass–graft surgery (38).

The evidences above well support the use of pEXOs and corresponding microRNAs to depict the extent of LV Rev–Rem in our MR patients during the late postoperative period. Since some reports have focused on their predictive value in dogs with MR (49) and in patients undergoing transcatheter valve repair (31), further investigation of their long-lasting cardioprotective role after early surgical valve repair is required.

In this study, we hypothesized that the postoperative change in the levels of pEXOs delivering specific microRNAs may underlie LV Rev–Rem in the primary MR patients after the early surgical MVR. For this purpose, we also used CMRI to better assess the late postoperative changes in cardiac structure and function. Using a gold standard approach, we measured both levels of pEXOs and exosomal microRNAs specifically related to remodeling and heart failure, such as miR-1, miR-133a, and miR208a (50–52), and to cardioprotection, such as miR-21-5p (53). Finally, the anti-remodeling properties of late pEXOs and those of selected exosomal miR were tested in cultured adult murine cardiomyocytes (HL-1) exposed to angiotensin II, an established *in vitro* model of cardiac hypertrophy (54, 55).

## Materials and methods

### Study design and patients

Our study was approved by Ethics Committee of “G. Monasterio” Foundation (FTGM, Massa, Italy) (EMIGRATE study, approval n°1529) in accord with the principles outlined in the Declaration of Helsinki. We obtained signed informed consent from each patient. Aiming to select patients with higher mitral valve repair probability, the inclusion criteria were as follows: Age (35–75 years); presence of severe primary MR due to prolapse or flail leaflet as assessed by transthoracic echocardiography, sinus rhythm, and clinical indication to surgery. According to clinical guidelines, severe primary MR was diagnosed when at least one of the following parameters was detected by transthoracic echocardiography (56–58): (1) Vena contracta width more than or 7 mm from the parasternal long-axis view, (2) effective regurgitant orifice (ERO) area more than or 0.4 cm^2^, as evaluated by proximal isovelocity surface area (PISA) method, and (3) regurgitant volume more than or 60 ml. The patients with echocardiography evidence of leaflet tethering (typical of secondary MR) or calcific degenerative restricted motion of leaflets were excluded. In the case that, during surgery, the surgeon decided to convert intervention to valve replacement, the patients were then excluded from the study. Other exclusion criteria comprehended the following: chronic kidney disease (CKD) defined as glomerular filtration rate less than 50 ml/m^2^, the previous cardiac surgery or history of congenital heart disease, the current or past myocardial ischemia/severe coronary artery disease, LV ejection fraction (LVEF) less than 40%, other-than-mitral valve diseases more than mild, and any contraindications to CMRI. The current cardiovascular risk factors, functional capacity according to the New York Heart Association (NYHA) classification (59), presence of CKD, and medications were assessed prior to surgery.

The complete clinical profile of patients is described in Table 1. Eight healthy Caucasian volunteers (age, <60 years) with no evidence of cardiac disease were recruited as a control group. Since we detected prevalence of males among our patients (Table 1), we enrolled only male volunteers to avoid gender bias.

**TABLE 1:** Clinical features and CMRI parameters of MR patients enrolled in the study.

Clinical picture	
Age (years)	55.2 ± 8.0 (50–69, 57.9%; 70+, 10.5%)
Sex (male)	94,74%
BMI (kg/m^2^)	26.1 ± 2.9 (25–30, 57.9%; 30+, 10.5%)
Smoking status (smoker)	15.8%
Hypertension	47.4%
Hypercholesterolemia	31.6%
Peripheral vascular disease	5.3%
Prior stroke/TIA events	0%
Heart failure	0%
Family history of CVD	21.1%
NYHA	I, 36.8%; II, 57.9%; III, 5.3%
CHA_2_DS_2_-VASc Score	0, 52.6%; 1, 31.6%; 2, 15.8%
Diabetes mellitus	0%
CKD	0%
**Pharmacotherapy**	
β-Blockers	84.2%
Ace-inhibitors	26.3%
Calcium antagonists	0%
Diuretics	63.2%
**Levels in blood**	
BNP (ng/L)	39.4 ± 34.4
K (mEq/L)	3.96 ± 0.19
Ca (mg/dl)	8.43 ± 0.32
Mg (mg/dl)	1.78 ± 0.23
**MR and surgery**	
Type of MR	Flail, 36.8%; Prolapse, 63.2%
Leaflet failure	Anterior, 5.3%; Posterior, 68.4%; Both, 21.1%
Surgical access	Right minithoracotomy, 94.7%; Median sternotomy, 5.3%
Implanted ring	CG Future band, 47.4%; Simulus semi-rigid, 47.4%; Profile 3D, 5.3%
*N* of neochords implanted	0, 22.2%; 1, 16.7%; 2, 61.1%
**Preoperative CMRI**	
LVEDVi (ml/m^2^)	113.67 ± 25.37
LVESVi (ml/m^2^)	42.50 ± 11.77
RVEDVi (ml/m^2^)	84.78 ± 15.57
RVESVi (ml/m^2^)	34.44 ± 9.00
LV Mass index (g/m^2^)	77.44 ± 13.36
LVSV (ml)	142.33 ± 35.93
LVSVi (ml/m^2^)	72.36 ± 16.98
RVSV (ml)	100.67 ± 20.93
RVSVi (ml/m^2^)	51.15 ± 9.72
LVEF (%)	62.60 ± 5.87
RVEF (%])	59.64 ± 6.56
Heart rate (BPM)	61.00 ± 7.92
Cardiac index (ml/min ⋅ m^2^)	4180.36 ± 1114.27
LA Area index (cm)	19.37 ± 7.89
RA Area (cm)	14.32 ± 5.71
LA-max volume index (ml/m^2^)	77.53 ± 27.15
LAEF (%)	53.44 ± 9.48
LVSV phase contrast (ml)	85 ± 19
Regurgitant volume (ml)	55 ± 31
Regurgitant fraction (%)	37.09 ± 14.00
LGE presence (*n*, %)	44.44%

Clinical features of N = 19 patients undergoing MVR surgery were evaluated prior to the surgical procedure; CMRI performed before (T0) the surgical procedure were also analyzed and are shown in the table. Categorical variables are reported as percentage; continuous variables are presented as mean ± SD.

We performed combined CMRI and blood sampling for pEXO isolation before surgery (baseline) and at 6 months after surgery. The timing of the experimental protocol was in accord with the previous studies (60, 61). Only patients who completed the whole protocol were included in the study.

### Mitral valve repair surgery and experimental protocol

All patients underwent same anesthetic protocol and standard hypothermic cardiopulmonary bypass (CPB) procedure. Successful surgical repair of the mitral valve was performed mainly through a minimally invasive endoscopic approach to the mitral valve (39, 62). Briefly, chest access was *via* a small right incision in the third or fourth intercostal space—a periareolar (in males) access or axillary (in females) minithoracotomy was performed. The median sternotomy was the alternative surgical approach when the minimally invasive approach was not possible (Table 1). An extracorporeal circulation was established through peripheral femoro–femoral cannulation using surgical cut-down approach. After opening the pericardium, the ascending aorta was gently cross-clamped externally and cold crystalloid cardioplegic solution (Custodiol) was infused in an antegrade fashion. The mitral valve was exposed through opening of the interatrial groove and the atrial lift retractor (USB Medical) was positioned exposing the mitral valve. The repair surgery was carried out according to the usual techniques in accord with the type of mitral pathology. All patients underwent implantation of a prosthetic annulus, and most also underwent the implantation of Gore-Tex prosthetic cords to restore proper posterior leaflet height (Table 1). The successful repair was assessed with intraoperative transesophageal echocardiography before suturing the surgical access. Cardiac magnetic resonance imaging and blood collection were performed prior to surgery (T0) and 6-months after surgery (T1) in awaken patients.

### Transthoracic echocardiography

Transthoracic echocardiography was performed with a commercial machine equipped with a 5–1 mHz phased array probe (iE33 system, Phillips Medical Systems, Andover, MA, United States). Left ventricular ejection fraction was evaluated with the modified Simpson Biplane method. The postoperative residual mitral regurgitation was evaluated using a scale from 0 (nil) to 4 (severe), in all patients. All the measurements were performed according to the current standards (63, 64).

### Cardiac magnetic resonance imaging

Cardiac magnetic resonance imaging (3.0T scanner, Ingenia, Philips) was used to assess cardiac remodeling and valve function. Briefly, endocardial and epicardial borders of both ventricles and left atrium (LA) were manually delineated at end-diastole (ED) and end-systole (ES) by an experienced operator. For both ventricles, ED and ES volumes (EDV and ESV, respectively) were calculated from a stack of cine balanced steady-state free precession (bSSFP) short-axis images acquired orthogonally to the LV long axis, from mitral valve to apex, without gap between each slice. Left ventricular myocardial mass was calculated by subtracting the total endocardial volume from the total epicardial volume then multiplying for myocardial density (1.06 g/ml). Left ventricular and right ventricular (RV) stroke volumes (LVSV and RVSV, respectively) were calculated as EDV– ESV. The body–surface area indexed values (*i*) were calculated for all parameters (65). To determine global cardiac function, ejection fraction (EF) was calculated as


(SV/EDV)×100

for both ventricles.

To assess the positive LV reverse remodeling following surgery, the reduction in LVEDV from T0 to T1 was computed as


(LVEDV⁢T1-LVEDV⁢T0)/LVEDV⁢T1

The patients with a percentage of change lower than the median value were included in the *High LV reverse remodeling group* (HiR-REM) while patients with values above the median value were included in the *Low LV reverse remodeling group* (LoR-REM).

Phase contrast sequences at the level of aorta and pulmonary artery roots were used to obtain a direct quantification of the blood volume pumped out of LV and RV at each systole, respectively (measured stroke volume). For each patient, the velocity of encoding was adapted to be the lower possible level that avoided aliasing noise. Regurgitant volume was obtained by subtracting the anterograde LVSV measured by the analysis of velocity encoded phase contrast images at the aortic root level (66). Briefly, once flow/time curve was generated, the anterograde SV was measured as the area under the curve of positive flow after the subtraction of the area under the retrograde flow in case of aortic regurgitation.

LVSV was also measured in short axis cine bSSFP as


LVEDV-LVESV⁢(66,)

and the mitral valve regurgitant volume was measured as


LVSV-anterograde⁢SV⁢(66,67)

The myocardial fibrosis was assessed by Late Gadolinium Enhancement (LGE) technique (68). Briefly, 10 min after intravenous injection of a gadolinium-based contrast agent [either Omniscan (GE Healthcare, Amersham, United Kingdom) or Magnevist (Schering, Berlin, Germany) at a concentration of 0.1–0.2 mmol/kg], a stack of short axis sections of both ventricles perpendicular to the LV long axis were acquired without gap using a T1-weighted gradient echo inversion recovery sequence. Appropriate Inversion time to null normal myocardium was chosen using a look-locker TI-scout. Slice thickness was set at 8 mm in all scans.

### Isolation of plasma exosomes

A 12-ml volume of peripheral venous EDTA-treated blood was collected from MR patients and controls. Plasma was isolated with a 10,000*g* centrifugation for 15 min at 10°C and was stored at –80°C until analysis. pEXOs were isolated and purified from 1-ml plasma using an optimized 2-d ultracentrifugation protocol [adapted from the study discussed in Ref. 39]. Briefly, plasma was diluted with 15 volumes of filtered (0.2-μm pore) phosphate buffer solution (PBS) and centrifuged at 3,000*g* for 15 min at 10°C. Supernatant was filtered (0.2-μm pore) and subjected to the following centrifugation series: 10,000*g* for 15 min at 10°C (discarding pellet), 20,000*g* for 30 min at 10°C (discarding pellet), then 100,000*g* for 5 h at 4°C. The pellet from the ultracentrifugation was re-suspended in 1 ml of PBS and stored at 4°C overnight. The day after, it was vortexed thoroughly and centrifuged at 10,000*g* for 15 min at 10°C. The supernatant obtained was diluted with 15 volumes of filtered PBS, and centrifuged at 20,000*g* for 30 min at 10°C. Eventually, the supernatant was ultracentrifuged at 100,000*g* for 5 h at 4°C. The final exosomal pellet was re-suspended in 100 μl of cold filtered PBS and was stored at –80°C until analysis.

### Quantification of plasma exosomes

The particle size and concentration were measured by nanoparticle tracking analysis (NTA) using a NanoSight LM10 NTA instrument with NTA 3.2 software for data acquisition and analysis (Malvern Panalytical). The pEXOs samples were diluted with filtered PBS to match the recommended particle concentration range {20–120 particles/field; [(1 × 10^7^)–(1 × 10^9^)] particles/ml}. Three 60-s videos were acquired for each sample maintaining a camera level, 10; and the videos were analyzed at detection threshold, 3. The modal particle size and particles concentration/ml was measured by the software. The pEXOs concentration was inferred from the particle size distribution up to 150-nm size, it was normalized by resuspension volume and expressed relative to the starting plasma volume.

### Western blot analysis of plasma exosomes

The analysis of exosomal proteins is used to characterize the profile of pEXOs in hospital setting (39). Isolated pEXOs were suspended in 100 μl RIPA buffer [50-mM Tris, 300-mM NaCl, 5-mM EDTA, 1% (v/v) NP40, 0.1% (w/v) SDS, 0.5% (w/v) di sodium deoxycholate] containing protease inhibitors (Sigma), incubated on ice for 30 min and then sonicated on ice for 5 min to improve homogenization efficiency. The protein concentration was measured using Pierce™ BCA Protein Assay Kit (Thermo Fisher Scientific) as previously described by us (39). Equal amounts of proteins (50 μg) were resolved by 12% SDS polyacrylamide gel and transferred to nitrocellulose membrane (Bio-Rad). Equal loading was controlled by Ponceau staining. The membranes were blocked with 5% (w/v) non-fat milk in TBST [TBS pH 7.4 containing 0.1% (v/v) tween-20] for an hour at room temperature. The primary antibodies were diluted in blocking buffer and membranes incubated overnight at 4°C to detect tetraspanins CD63 (1:1,000, anti-CD63, Santa Cruz Biotechnology) and CD81 (1:1,000, anti-CD81, Santa Cruz Biotechnologies), and tumor susceptibility gene 101 (1:1,000, anti-TSG101, Sigma) common hallmarks of human pEXOs (44, 69). Specific protein bands were detected by chemiluminescence (ECL substrate, Thermo Fisher Scientific) after incubation with a goat horseradish peroxidase-conjugated antibody toward either rabbit or mouse IgGs (1:3,000 dilution in blocking buffer; Sigma) for 2 h at room temperature. Densitometry analysis of protein bands was performed using ImageJ software (National Institute of Health, United States) as previously showed by us (39).

### The quantitative reverse transcription PCR analysis

To quantify microRNA expression on the exosomes, RNA was isolated using miRNeasy mini kit for cells and tissues (Qiagen) following the manufacturer’s guidelines with few adaptations. Briefly, the isolated pEXOs were lysed by incubation with 5 volumes QIAzol reagent for 5 min, then 5 μl of 0.33-nM CelMir39 spike-in control (1.65 fmol) and 1 μL of 20 μg/μL molecular grade mussels’ glycogen (Sigma) were added to each sample, followed by one volume of chloroform. The samples were vortexed briefly, incubated for 3 min at room temperature and centrifuged at 12,000*g* for 15 min at 4°C. The upper aqueous phase containing the RNA was collected and mixed with 1.5 volumes of 100% (v/v) ethanol. Each sample was transferred into RNeasy^®^ Mini columns (Qiagen). The RNA binding, washed and eluted according to the manufacturer’s instructions. Moreover, RNA was eluted in 30 μl of RNAse-free water. Template RNA (7 μl) was reverse transcribed using the miScript II Reverse Transcription (RT) kit (Qiagen) on a 20 μl final volume in line with the manufacturer’s instructions. Also, miScript HiSpec Buffer was used in each reaction to selectively target the mature miRNA forms. Before proceeding to quantitative PCR, the cDNA was diluted 12.5 times in nuclease free water. The quantitative PCR was carried out on 5-μl diluted cDNA using the QuantiTect SYBR Green PCR reaction mix (Qiagen) on a 20-μl final reaction volume according to the manufacturer’s instructions. Forward primers for each selected miR were inferred from mature miRNA sequences and purchased from Sigma (Table 2). The miScript Universal Primer (Qiagen) was utilized as a reverse primer for all reactions. All primers were employed at 500-μM concentration. The reaction was performed on a Rotor-Gene Q real-time PCR cycler (Qiagen) at the following thermocycling conditions: 15 min at 95°C to activate HotStarTaq DNA Polymerase followed by 40 thermo cycles composed of 15 s at 94°C, 30 s at 55°C, and 30 s at 70°C. The relative quantification of miR expression was performed by applying the 2^–ΔΔCt^ comparative method (70). For each patient, CelmiR-39 was employed as the housekeeping gene in the calculation of ΔCt, to normalize miR expression to RNA recovery. For each miRNA analyzed, the average ΔCt of all control patients acted as a calibrator for calculating the ΔΔCt. The resulting 2^–ΔΔCt^ data represent the relative expression of the given miRNA compared to the healthy controls.

**TABLE 2:** Mature miRNA sequences analyzed in this study and related forward primers employed for qPCR. All primers were obtained from Sigma and employed at 500-μM concentration.

	Mature miRNA (Sequence 5′-3′)	Forward primer (5′-3′)
miR-1	hsa-miR-1-3p (UGGAAUGUAAAGAAGUAUGUAU)	TGGAATGTAAAGAAGTATGTAT
miR-21	hsa-miR-21-5p (UAGCUUAUCAGACUGAUGUUGA)	TAGCTTATCAGACTGATGTTGA
miR-133a	hsa-miR-133a-3p (UUUGGUCCCCUUCAACCAGCUG)	TTTGGTCCCCTTCAACCAGCTG
miR-208a	hsa-miR-208a-3p (AUAAGACGAGCAAAAAGCUUGU)	ATAAGACGAGCAAAAAGCTTGT
Cel-miR-39 (Housekeeping spike-in gene)	cel-miR-39-3p (UCACCGGGUGUAAAUCAGCUUG)	TCACCGGGTGTAAATCAGCTTG

To assess mRNA expression on cultured cells, RNA was isolated using PRImeZOL™ reagent (Canvax Reagents SL), following the manufacturer’s instructions. Furthermore, RNA was re-suspended into 40 μl of RNAse-free water and quantified by NanoDrop spectrophotometry (Thermo Fisher Scientific); 1 μg of the total RNA was reverse transcribed with the PrimeScript RT kit (Takara) on a 20-μl final volume, following manufacturer’s guidelines. The cDNA obtained was diluted 1:10 and analyzed by quantitative PCR using the TB Green^®^ Premix Ex Taq™ reagent (Takara). The following primers, obtained from Sigma, were employed in the analysis at 500-μM concentration: Atrial natriuretic peptide (ANP) 5′-TCGTCTTGGCCTTTTGGCTT-3′ (forward) and 5′-AGGTGGTCTAGCAGGTTCTTGAAA-3′ (reverse); Brain natriuretic peptide (BNP): 5′-CGTTTGGGCTGTAACGCACT-3′ (forward) and 5′-TCACTTCAAAGGTGGTCCCAG-3′ (reverse); Cardiac β-myosin, β-MHC (MYH7) 5′-TCCTGCTGTTTCCTTACTTGCT-3′ (forward) and 5′-GCTGAGCCTTGGATTCTCAAAC-3′ (reverse); Sarcoplasmic/endoplasmic reticulum Ca^2+^-ATPase 2a (SERCA2a) 5′-CCTTTGCCGCTCATTTTCCAG-3′ (forward) and 5′-GGCTGCACACACTCTTTACC-3′ (reverse); β-Actin 5′-GGCACCACACCTTCTACAATG-3′ (forward) and 5′-GGGGTGTTGAAGGTCTCAAAC-3′ (reverse). All reactions were performed on a Rotor-Gene Q real-time PCR cycler (Qiagen) at the following thermocycling conditions: 30 s at 95°C followed by 40 cycles of 15 s at 95°C and 30 s at 60°C as suggested by the manufacturer’s guidelines. The relative quantification of mRNA expression was performed by applying the 2^–ΔΔCt^ comparative method (68), with β-Actin employed as the housekeeping gene.

### Cell culture and experimental protocol

Murine cardiomyocyte cell line HL-1 (a kind gift of W. C. Claycomb, Louisiana State University, New Orleans, LA, United States) was used to evaluate anti-hypertrophic role of human pEXOs. The HL-1 cells were cultured in Claycomb Medium (71) (Sigma) supplemented with 10% (v/v) fetal bovine serum (Sigma), 100-μM norepinephrine, 100 units/ml of penicillin, 100-μg/ml streptomycin, 250-ng/ml Amphotericin B and 2-mM L-Glutamine (Sigma) at 37°C in 5% CO_2_. For the experimental purposes, HL-1 cells were seeded at a concentration of 5,000 cells/well on fibronectin pre-coated (1.25-μg/cm^2^ human fibronectin, Sigma; 1 h at 37°C) 8-well chamber slides (Milllicell EZ, Millipore) and let adhere for 24 h before the treatment. Cardiomyocytes were then exposed to 1-μM angiotensin II (AngII; Sigma) in complete Claycomb medium for 48 h (54) (antibody-free complete Claycomb medium was employed in case of subsequent miRNA inhibition). T0- or T1-pEXOs (1 × 10^9^ particles/ml) isolated from each group of patients or miRNA inhibitors (30 nM) were added 24 h after the beginning of AngII treatment, and the treatment was maintained for the remaining 24 h. Sterile filtered (0.2-μm pore) PBS vehicle was employed as a negative control.

### MiR-21-5p inhibition and plasma exosomes

Furthermore, MiRVANA miRNA miR-21-5p inhibitor and miRVANA miRNA inhibitor negative control #1 were purchased from Thermo Fisher Scientific. To perform direct miR-21-5p inhibition in HL-1 cardiomyocytes, cells were transfected with 30 nM miRNA-21 inhibitor/negative control using Lipofectamine^®^ RNAiMAX Transfection Reagent (3-μl/ml culture medium; Thermo Fisher Scientific) for 24 h, following manufacturer’s instruction. All procedures were performed in antibody-free Claycomb medium.

As a different approach, isolated pEXOs were pre-loaded with miR-21 inhibitor then added to HL-1 cells as described above. The protocol for oligonucleotides loading was adapted from Zhang et al. (72). Briefly, 200-pmol miRNA miR-21-5p inhibitor/negative control was combined with pEXOs (5 × 10^8^ particles in PBS), then 30-μl of 1-M CaCl_2_ were added, and the final volume was adjusted to 300 μl with PBS. The mixture was incubated on ice for 30 min, then heat shocked for 60 s at 42°C and placed again on ice for 5 min. Treated pEXOs were then collected again by differential centrifugation as follows: After a 15-min clear-up step at 10,000*g* at 4°C, exosomes were isolated with a 5-h ultracentrifugation at 100,000*g*, performed at 4°C. The pEXOs collected (approximately 5 × 10^8^ particles) were then re-suspended in 100 uL PBS. The amount of pEXOs obtained with this procedure is sufficient to treat HL-1 cardiomyocytes in one 8-well chamber slide well, in a final volume of 500 μl (1 × 10^9^ particles/ml). All procedures were again performed in antibody-free Claycomb medium.

### Measurement of HL-1 size

Actin filaments were stained with phalloidin to define HL-1 cell area (73). Briefly, the cell monolayer was washed twice with PBS for 5 min, fixed with 4% (w/v) paraformaldehyde (PFA) in PBS (Sigma) for 15 min and permeabilized with 0.1% (v/v) TritonX-100 (Sigma) in PBS twice for 2 min, at room temperature; PBS washes (2 × 5 min) were performed after each step. Cells were incubated with Phalloidin-Atto 550 (1:300; Sigma) in PBS for 1 h at room temperature to stain cytoskeletal F-actin. 4′,6-Diamidino-2-phenylindole dihydrochloride solution (DAPI; 1:1,000, Sigma) was also added to the solution to stain the nuclei. Excess dye was removed by PBS wash (2 × 5 min) and the slide was mounted with Aqua-Poly/Mount aqueous mounting medium (Polysciences, Inc.). Images were acquired at a fluorescence optical microscope (DM 2500, Leica Microsystems, Germany) (20× magnification). The cell area was measured manually with ImageJ software^1^ and expressed as pixels.

### Statistical analysis

Continuous clinical variables and CMRI measurements are presented as mean ± SD. Categorical clinical variables are presented as percentages of the total. The pEXOs concentration and miRNA expression are presented as median with interquartile range (IQR). Fisher’s exact test was used to reveal differences between the frequencies of categorical variables. Regarding continuous variables, normal distribution was evaluated by the Kolmogorov-Smirnov test. Homoscedasticity was evaluated by the *F*-test of equality of variances (2 groups) or the Brown–Forsythe test (more than 2 groups). Robust Regression and OUTlier removal (ROUT) method was employed to identify outliers (Q = 0.1, FDR < 0.1%), which are reported in the graphs as “×” symbols. Paired Student’s *t*-test or the Wilcoxon test were applied to identify significant differences between repeated measurements according to their distribution. Independent samples Student’s *t*-test (with or without Welch’s correction, depending on homoscedasticity) or the Mann–Whitney test were employed between two independent groups according to their distribution. One-way ANOVA (or Brown–Forsythe and ANOVA test, depending on homoscedasticity) or the Kruskal–Wallis test, depending on sample distribution, were applied when more than two groups were compared. All statistical tests were performed as two-sided, statistical significance was considered for *p* < 0.05.

Receivers operating characteristic (ROC) curves were constructed with easyROC open source online software (74) using each pEXOs or each corresponding microRNA expression value. The area under the curve (AUC) with 95% CI was calculated for each ROC curve; standard errors and confidence intervals were estimated by applying the DeLong method (75). The Wald test was used to check the null hypothesis that the AUC is equal to 0.5 (i.e., no predictive power).

## Results

### Characteristics of patients

During a 1-year timeframe (from January 2018 to February 2019), 23 surgical MR patients met inclusion criteria and were enrolled in the study. All the patients underwent to successful MVR. The time of extracorporeal circulation was 134 (114–166) min and the cross-clamp time was 84 (75–101) min; all surgeries were carried out through the initial incision with no need for sternotomy conversion. We had no cases of conversion to replacement during intervention. Four patients were excluded after surgery because one patient died early after the intervention and three patients voluntarily withdrew from the study. Therefore, the final population consisted of 19 patients. As shown in Table 1, 19 patients, with average age 55.2 ± 8.0 years, showed average mitral regurgitant volume of 55 ± 32 ml, average regurgitant fraction of 37.09 ± 14.00 % and a high incidence of mitral valve prolapse (63.2%), with predominant involvement of the posterior leaflet (68.4%). Most of the patients were males, 36.8% in NYHA class I, 57.9% in NYHA class II and 5.3% in NYHA class III, and had low atrial fibrillation-associated stroke risk (CHA_2_DS_2_-VASc Score = 0, 52.6%; Table 1). More than 20% patients had history of cardiovascular diseases in their family and 15.8% were smokers (Table 1). Hypertension was the main primary comorbidity among the MR patients (47.4%), followed by hypercholesterolemia (31.6%) and peripheral vascular disease (5.3%). No patient had prior ischemic events (Table 1). Interestingly, cardiac fibrosis was detected in less than 50% of patients as measured by CMRI (Table 1). Most patients underwent MVR through right minithoracotomy (94.7%) and were mainly treated with beta-blockers (84.6%), diuretics (63.2%), and angiotensin-converting enzyme (ACE) inhibitors (26.2%) before surgery (Table 1). Postoperative atrial fibrillation occurred in 7 patients (roughly 37%; LoR-REM, *n* = 5; HiR-REM, *n* = 2). No other postoperative complications were observed in the study population. All patients underwent to a transthoracic echocardiography within 1 month after surgery, showing a good postsurgical outcome with nil (grade 0/4; 5/9 LoR-REM; 6/10 HiR-REM) or mild (grade 1/4; 4/9 LoR-REM; 4/10 HiR-REM) residual mitral regurgitation and preserved LV EF (LoR-REM, 58.6 ± 10%; HiR-REM, 59.12 ± 9%).

### Perioperative cardiac assessment by cardiac magnetic resonance imaging

**FIGURE 1:**
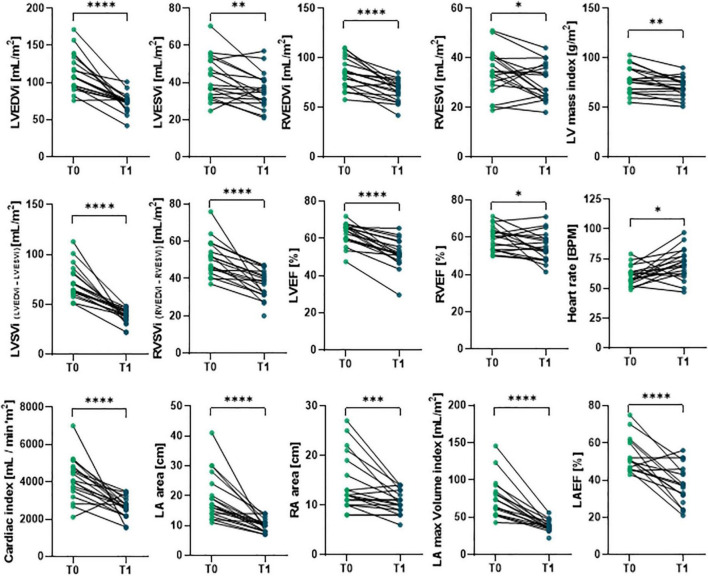
Comparison of preoperative and postoperative CMRI results in primary MR patients undergoing MVR surgery. The CMRI was performed on patients undergoing MVR surgery (*n* = 19) before (T0) and 6 months after (T1) surgery. Individual data are shown for each patient at both time points; statistical comparison between T0 and T1 was performed by paired Student’s *t*-test or the Wilcoxon test depending on data distribution. *p < 0.05; ^**^p < 0.01; ^***^p < 0.001; ^****^p < 0.0001.

Cardiac magnetic resonance imaging was performed before (T0) and 6-months (T1) after surgery to assess cardiac phenotype in all patients (Figure 1 and Supplementary Table 1). Hallmarks of LV Rev–Rem, indeed, were detectable in most patients at 6 months after MVR. In particular, postoperative LV end-diastolic volume index (LVEDVi) was significantly reduced to more than 10% of the pre-surgical value in almost all patients (*p* = 1.90 × 10^–6^; Figure 1 and Supplementary Table 1). Similarly, LV end-systolic volume index (LVESVi; *p* = 0.0055), RV end-diastolic volume index (RVEDVi; *p* = 1.30 × 10^–5^) and RV end-systolic volume index RVESVi (*p* = 0.035) were lower at T1 compared to T0 (Figure 1 and Supplementary Table 1). The LA area (*p* = 7.60 × 10^–6^) and volume (*p* = 1.30 × 10^–5^), as well as RA area (*p* = 0.001), were reduced in accord with the previous findings (76) (Figure 1 and Supplementary Table 1). Indexed LV mass was also decreased after surgery (*p* = 0.0035; Figure 1 and Supplementary Table 1). Of note, LV and RV EF were significantly reduced at 6-months after the surgery compared to T0 (*p* = 2.80 × 10^–7^ and *p* = 0.0215, respectively), even if its preoperative values were higher than 50% for most patients (Figure 1 and Supplementary Table 1). Similarly, both LV and RV stroke volume indexes at T1 were reduced as compared to preoperative values (*p* = 7.26 × 10^–7^ and 3.70 × 10^–5^, respectively). Postoperative cardiac index values were also lower than ones at T0 (*p* = 7.40 × 10^–5^), which remained within physiological range; while higher resting heart rate was observed after surgery (*p* = 0.01; Figure 1 and Supplementary Table 1).

### Role of preoperative cardiac phenotype to predict the extent of left ventricular reverse remodeling after surgery

**FIGURE 2:**
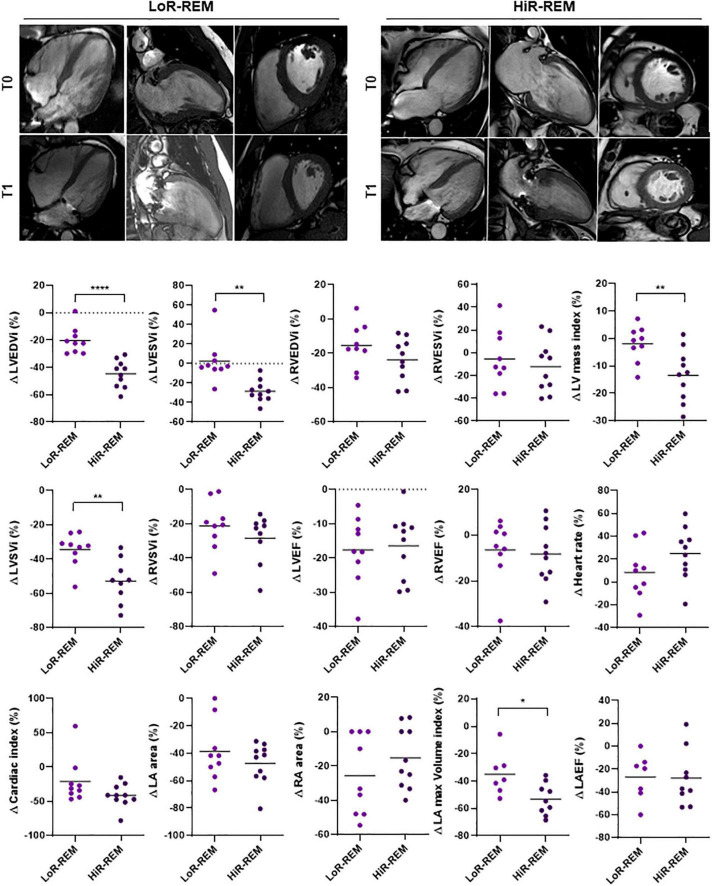
Analysis of CMRI parameters change in MR patients with different grade of reverse remodeling after MVR surgery. The CMRI data were collected from patients undergoing MVR surgery (*n* = 19) before (T0) and 6 months after (T1) surgery. The CMRI acquisitions are shown for a representative Low LV reverse remodeling (LoR-REM) and High LV reverse remodeling patient (HiR-REM). For each CMRI parameter measured, the percentage change between T0 and T1 was computed for each patient as %Δ = (T1 – T0)/T0 × 100. The data are compared between LoR-REM and HiR-REM patients. Individual data are shown; horizontal bars represent group average. Statistical significance of the differences was evaluated by independent samples Student’s *t*-test (with or without Welch’s correction) or the Mann–Whitney test depending on data distribution. *p < 0.05; **p < 0.01; ****p < 0.0001.

The extent of the postoperative LV reverse remodeling was assessed for each patient by calculating the percentage change (%Δ) of LVEDVi at T1 compared to T0 using a cut-off value of –30% (Figure 2). Patients with high degree of LV Rev–Rem showed %Δ LVEDVi values significantly reduced by 44.61 ± 9.94% (HiR-REM group, *n* = 10); otherwise, patients with low degree of LV Rev–Rem showed %Δ LVEDVi values significantly reduced by 20.23 ± 9.87% (LoR-REM, *n* = 9; *p* = 5.20 × 10^–5^; Figure 2 and Supplementary Table 2). Of note, HiR-REM patients were characterized by preoperative LVEDVi higher than LoR-REM group (*p* = 0.021; Supplementary Figures 1, 2).

To evaluate the role of preoperative cardiac parameters to predict postoperative LV reverse remodeling, we first compared the clinical and CMRI-derived features at T0 in HiR-REM and LoR-REM groups. We found that both groups have similar clinical profile (Table 3). Moreover, most of HiR-REM patients were in NYHA class I (60%; Table 3).

**TABLE 3:** Analysis of preoperative clinical parameters in MR patients with different grade of reverse remodeling after MVR surgery.

Preclinical picture	LoR-REM	HiR-REM		*p*
Age (years)	54.78 ± 7.81	55.60 ± 9.01	Ns	0.8351
BMI (kg/m^2^)	26.51 ± 3.05	25.75 ± 2.94	Ns	0.5884
Smoking status	11.11%	20.00%	Ns	>0.9999
Hypertension	55.56%	40.00%	Ns	0.6563
Hypercholest- erolemia	22.22%	40.00%	Ns	0.6285
Peripheral vascular disease	0.00%	10.00%	Ns	>0.9999
Family history of CVD	33.33%	10.00%	Ns	0.3034
NYHA	I, 11.11% II, 77.7% III, 11.11%	I, 60.00% II, 40.00% III, 0.00%	Ns	0.0573 for I
CHA_2_DS_2_-VASc Score	0, 44.44% 1, 44.44% 2, 11.11%	0, 60.00% 1, 20.00% 2, 20.00%	Ns	0.6563 for 0
β-Blockers	88.89%	80.00%	Ns	>0.9999
Ace-inhibitors	33.33%	20.00%	Ns	0.6285
Diuretics	66.67%	60.00%	Ns	>0.9999
BNP (ng/L)	36.13 ± 26.60	42.63 ± 44.50	Ns	>0.9999
K(mEq/L)	3.90 ± 0.21	4.02 ± 0.18	Ns	0.2706
Ca (mg/dl)	8.28 ± 0.32	8.55 ± 0.31	Ns	0.0953
Mg (mg/dl)	1.70 ± 0.12	1.85 ± 0.29	Ns	0.1563
Type of MR	Flail, 33.33% Prolapse, 66.67%	Flail, 40.00% Prolapse, 60.00%	Ns	>0.9999
Leaflet failure	Anterior, 11.11% Posterior, 66.67% Both, 22.22%	Anterior, 0% Posterior, 77.78% Both, 22.22%	Ns	>0.9999
LVSV phase contrast (ml)	80.22 ± 13.77	90.67 ± 22.82	Ns	0.2570
Regurgitant Volume (mL)	37.56 ± 16.30	73.67 ± 34.00	▲	0.0110
Regurgitant Fraction (%)	30.86 ± 10.38	43.32 ± 14.89	Ns	0.0561
LGE presence (*n*, %)	55.56%	33.33%	Ns	0.6372

Clinical picture of N = 19 patients undergoing MVR was evaluated prior to the surgical procedure (T0). Data are compared between LoR-REM and HiR-REM patients. Categorical variables are presented as percentage of the total; continuous variables are presented as mean ± SD. Statistical significance of the differences was evaluated by Fisher’s exact test for the categorical variables, while continuous variables were analyzed by independent samples Student’s t-test (with or without Welch’s correction) or the Mann–Whitney test depending on data distribution. The triangle means that it is a statistically significant increase.

Second, patients with greater preoperative mitral regurgitant volume (*p* = 0.011), LVEDVi (*p* = 0.021), indexed LA volume (*p* = 0.037), LVSVi (*p* = 0.0013) and cardiac index (*p* = 0.007) showed high level of postoperative Rev–Rem (Table 3, Supplementary Figure 1 and Supplementary Table 2). Interestingly, preoperative LVEF was similar in HiR- and LoR-REM patients (Supplementary Figure 1 and Supplementary Table 2).

Finally, the preoperative myocardial fibrosis assessed by CMRI was detectable in patients with primary MR (up to 40%) and even more in patients with mitral leaflets prolapse. Myocardial fibrosis is a hallmark known to be associated to adverse postsurgical outcomes and arrhythmic events (68, 77). In our study, myocardial fibrosis affected 44.4% of patients in the absence of ischemic pattern (Table 1).

### Role of perioperative plasma exosome levels to predict the extent of left ventricular reverse remodeling after surgery

**FIGURE 3:**
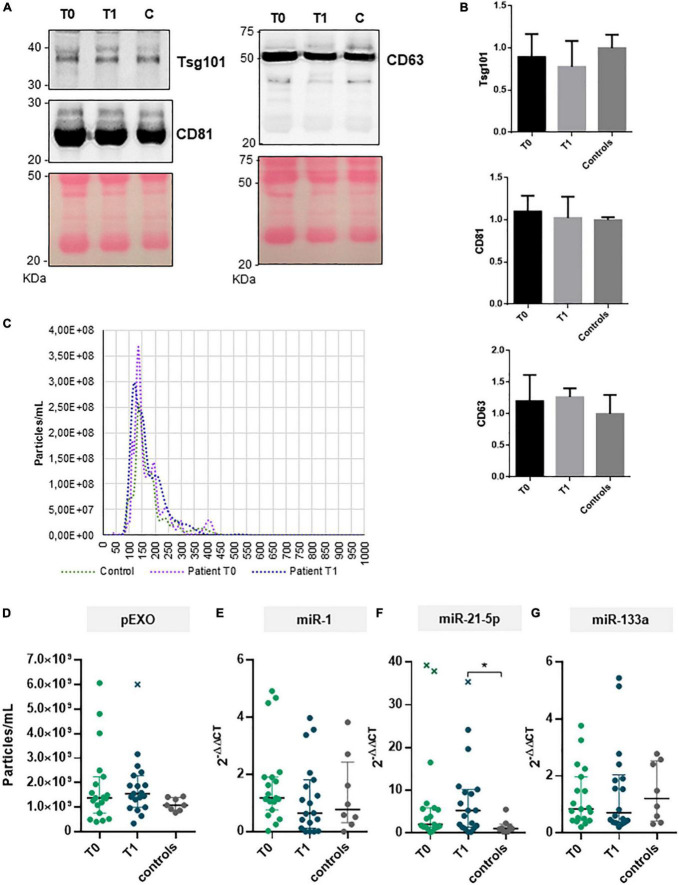
pEXOs and exosomal miRNAs in primary MR patients subjected to MVR surgery. The pEXOs were isolated from plasma of MR patients (*n* = 19) collected before (T0) and 6 months after MVR surgery (T1); pEXOs isolated from healthy controls (*n* = 8) were used as control. **(A,B)** The exosomal markers TSG101, CD81, and CD3 were assessed on the isolated particles by Western blot as described in the methods. **(A)** One representative experiment is shown. **(B)** Densitometric analysis of each protein target normalized for the corresponding experiment’s Ponceau staining shows no difference in marker expression among extracts (*N* = 3 patients/group). **(C)** Isolated pEXOs were analyzed by NTA as described in the methods; representative size distribution curves are shown. **(D)** The pEXOs concentrations were inferred by the NTA’s size distribution curve and shown as particles/ml of starting plasma. **(E–G)** Exosomal miRNA-1 **(E)**, miRNA-21-5p **(F)** and miRNA-133a **(G)** were assessed by qRT-PCR as described in the methods. Individual data, median value and interquartile range are shown in the graphs. Statistical significance of the differences between matching T0 and T1 data was evaluated by paired Student’s *t*-test or the Wilcoxon test depending on data distribution. Statistical differences between patients and controls were evaluated by one-way ANOVA (with or without Brown–Forsythe modification for heteroscedastic groups) or the Kruskal–Wallis test, depending on sample distribution; *p < 0.05.

In the current study, we isolated and characterized exosomes from plasma collected before and 6-months after MVR surgery. Plasma was also collected from healthy subjects (*N* = 8, control). Plasma exosomes (pEXOs) were positive for canonical markers CD63, CD81, and TSG101 (Figures 3A,B).

**FIGURE 4:**
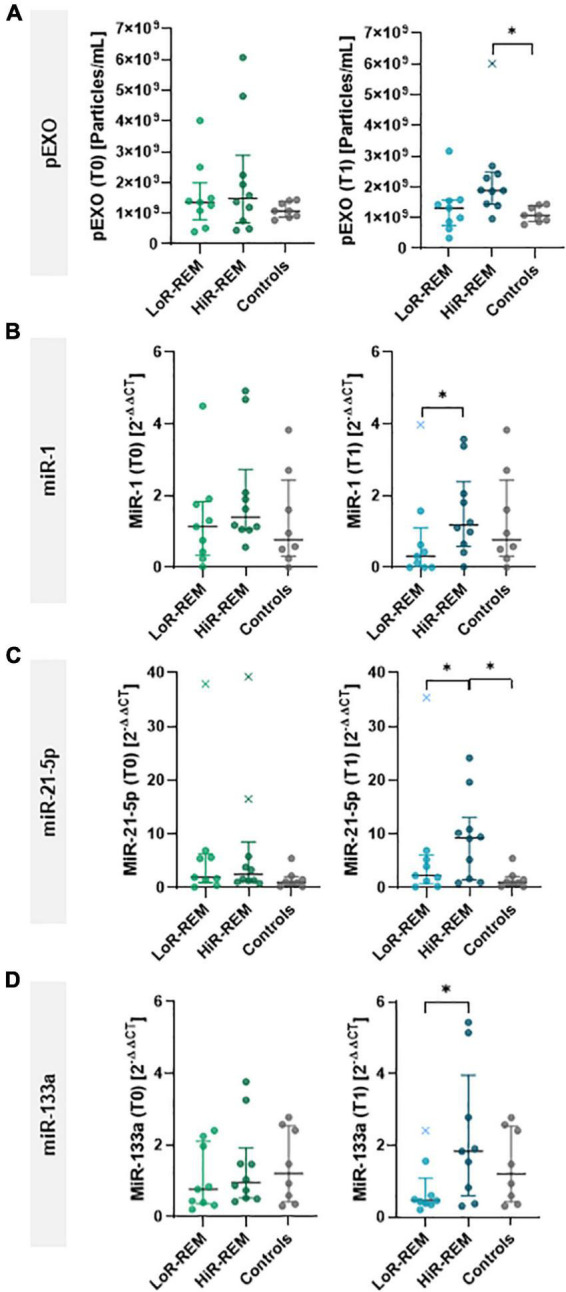
Analysis of pEXOs and exosomal miRNA in MR patients with different grade of reverse remodeling after MVR surgery. The pEXOs were isolated from plasma of MR patients (*n* = 19) collected before (T0) and 6 months after MVR surgery (T1); pEXOs isolated from healthy controls (*n* = 8) were used as control. **(A)** Isolated pEXOs were quantified by NTA as described in the methods. **(B–D)** Exosomal miRNA-1 **(B)**, miRNA-21-5p **(C)** and miRNA-133a **(D)** were quantified through qRT-PCR. Preoperative (T0) and postoperative (T1) levels were compared between LoR-REM and HiR-REM patients. Individual values are shown in the graphs; bars represent median and interquartile range. Statistical significance of the differences between patients’ groups was evaluated by independent samples Student’s *t*-test (with or without Welch’s correction) or the Mann–Whitney test depending on data distribution. Statistical differences between patients and controls were evaluated by one-way ANOVA (with or without Brown–Forsythe modification for heteroscedastic groups) or the Kruskal–Wallis test, depending on sample distribution; *p < 0.05.

All MR patients showed no difference in terms of preoperative pEXOs levels compared to the healthy subjects (median concentration 1.38 × 10^9^ particles/ml *vs* 1.08 × 10^9^ particles/ml, *p* = 0.381; Figures 3C,D). Similarly, no significant change in pEXO levels was observed 6 months after surgery (1.55 × 10^9^ particles/ml, *p* = 0.932 *vs* T0; *p* = 0.175 *vs* controls; Figure 3D). Conversely, when patients were grouped in terms of postsurgical LV remodeling, HiR-REM patients tended to have higher postoperative pEXOs levels (T1, 1.89 × 10^9^ particles/ml) than LoR-REM patients (T1, 1.31 × 10^9^ particles/ml, *p* = 0.062) and healthy subjects (1.08 × 10^9^ particles/ml, *p* = 0.016; Figure 4A and Supplementary Table 3). It is conceivable that pEXOs may contribute to late LV reverse remodeling after surgical mitral valve repair.

Therefore, we further assessed the prognostic value of pEXOs and we clustered patients according to the percentage change (%Δ) of pEXOs at T1 compared to T0. In 12 patients, pEXOs levels were significantly higher after surgery (+88% in average; median concentration 1.14 × 10^9^ particles/ml *vs* 1.70 × 10^9^ particles/ml, *p* = 0.015), while in seven patients the exosomal levels decreased significantly from T0 to T1 (–56% in average; median concentration 2.50 × 10^9^ particles/ml *vs.* 1.42 × 10^9^ particles/ml, *p* = 0.013; *p* = 3.97 × 10^–5^; Supplementary Figure 2). Patients with higher postoperative pEXOs levels showed a significant reduction of LV mass index from T0 to T1 in comparison to the group with a reduction of pEXOs after surgery (–11.62% *vs.* –1.75 %, *p* = 0.032; Supplementary Figure 2 and Supplementary Table 4).

**FIGURE 5:**
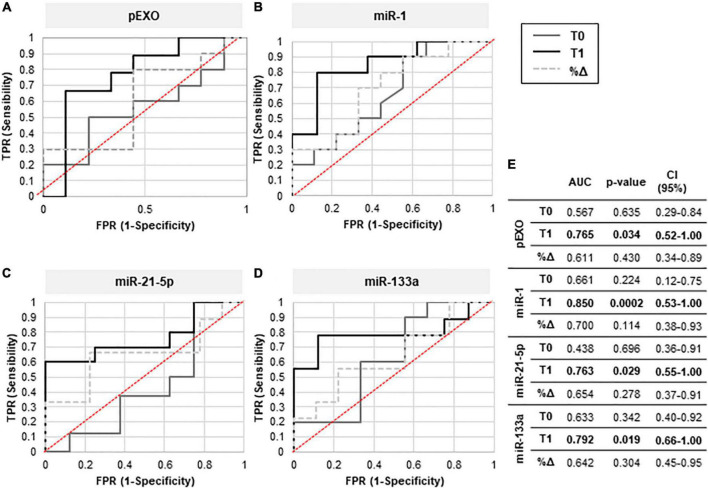
Diagnostic efficacy of pEXOs levels and exosomal miR-1, miR-21-5p, and miR-133a. The pEXOs were isolated from plasma of MR patients (*n* = 19) collected before (T0) and 6 months after MVR surgery (T1). **(A)** Isolated pEXOs were quantified by NTA as described in the methods. **(B–D)** Exosomal miRNA-1 **(B)**, miRNA-21-5p **(C)** and miRNA-133a **(D)** were quantified through qRT-PCR as described in the method. Patients were divided into HiR-REM and LoR-REM groups. The diagnostic efficiency of T0, T1, and %Δ levels of pEXOs (particles/uL) **(A)** and of the microRNAs miR-1 **(B)**, miR-21-5p **(C)** and miR-133a **(D)** (2^–ΔΔCT^) in distinguishing patients with Low and High postoperative reverse LV remodeling was evaluated by the receiver operating characteristic (ROC) curve analysis as described in the methods. The AUC quantification and statistical significance (*p*-value) are reported in the table **(E)**.

Receivers operating characteristic curves were constructed for T0-pEXOs and T1-pEXOs, as well as %Δ pEXO, from HiR- *vs.* LoR-REM (Figures 5A,E) to compare their diagnostic value to predict magnitude of LV reverse remodeling after surgical MVR. The AUC for T0-pEXOs was 0.567 (95% CI: 0.29–0.84; *p* = 0.635) and for T1-pEXOs was 0.765 (95% CI: 0.52–1.00; *p* = 0.034), indicating reliable predictive power of the postoperative pEXO levels (Figure 5A). Our data suggest that pEXOs levels detected at 6 months after surgery may be reliable enough to depict HiR- and LoR-REM patients.

### Relationship between exosomal miR-1, miR-21-5p, and miR-133a and left ventricular reverse remodeling

We further aimed to characterize some miRNAs packaged in postoperative pEXOs. Postoperative (T1) levels of exosomal miR-21-5p were higher in the entire MR population than in healthy controls (∼4-fold, *p* = 0.021), and tended to be higher than preoperative values (T0, ∼2-fold, *p* = 0.052; Figure 3F). In contrast, exosomal miR-1 and miR-133a levels did not change after surgery and were similar to healthy controls (Figures 3E,G). Interestingly, miR-208a was not detectable in the isolated pEXOs from the same patients.

Finally, we compared the levels of selected exosomal miRNAs between patients who experienced substantial postoperative reverse remodeling (HiR-REM patients) and patients with minimal reverse remodeling (LoR-REM patients). At T1, exosomal miR-21-5p levels in HiR-REM group were higher than those in the LoR-REM patients (3.1-fold, *p* = 0.030) and healthy subjects (3.1-fold, *p* = 0.025; Figure 4C and Supplementary Table 3). Although the perioperative cargo of exosomal miR-1 and miR-133a was tenfold lower than exosomal miR-21-5p, we found that the T1 levels of miR-1 and miR-133a were reduced in LoR-REM patients compared to HiR-REM by 2.8-fold (*p* = 0.018) and 3.0-fold (*p* = 0.046), respectively (Figures 4B,D and Supplementary Table 3). Interestingly, the levels of exosomal miR-1 and miR-133a were similar in both HiR-REM and control groups (Figures 4B,D and Supplementary Table 3).

As showed in Figures 5B,D,E, receiver operator characteristic (ROC) curves were constructed separately for miR-1, miR-21-5p, and miR-133a levels highlighting significant changes at T1 compared to T0, including %Δ miR-1, miR-21-5p, and miR-133a from HiR- *vs.* LoR-REM groups. The AUC for T1-miR1 was 0.85 (95% CI 0.53–1.00; *p* = 0.0002), for T1-miR-21-5p was 0.763 (95% CI 0.55–1.00; *p* = 0.029) and for T1-miR133a was 0.792 (95 % CI 0.66–1.00; *p* = 0.019) indicating fair predictive power at 6 months after MVR, Therefore, postoperative exosomal miR levels may be reliable enough to depict HiR- and LoR-REM patients.

### Anti-remodeling effects of exosomal miR-21-5p

**FIGURE 6:**
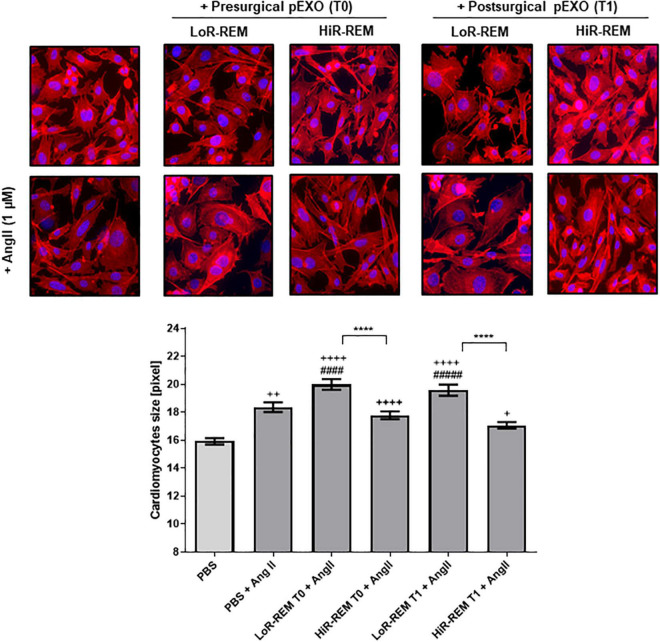
Effect of pEXOs from patients with different grade of reverse ventricular remodeling on cardiomyocytes size. The HL-1 cells were seeded at a concentration of 5,000 cells/cm^2^ and allowed to adhere for 24 h before treatment. To enlarge cardiomyocyte size, HL-1 cells were treated with ± 1-μM Angiotensin II (AngII, Sigma) in complete Claycomb medium for 48 h. A PBS suspension of pEXOs isolated at T0 or T1 from HiR- or LoR-REM group (*N* = 4 patients/group) was added to a final concentration of 1 × 10^9^ particles/ml, 24 h after the beginning of the treatment, and the treatment was maintained for the remaining 24 h; PBS was employed as a control. The cell monolayer was stained with Phalloidin-Atto 550 as described in the methods. The images were acquired with a fluorescence microscope (Leica; 20× magnification); the cell areas were manually measured with ImageJ software (https://imagej.nih.gov/ij/) and expressed in pixels. Statistical significance of the differences between treatments was evaluated by one-way ANOVA. The symbol “+” represents the significance of the differences against PBS alone (+, *p* < 0.05; ++, *p* < 0.01; ++++, *p* < 0.0001); “#” against PBS + AngII (^####^*p* < 0.0001); and “*” between LoR-REM and HiR-REM pEXO treatment in presence of AngII (****p < 0.0001).

*In vitro*, we have tested the anti-remodeling effects on murine cardiomyocyte of a single dose of pEXOs isolated from HiR-REM and LoR-REM patients. As showed in Figure 6, the preoperative (T0) and postoperative (T1) pEXOs isolated from LoR-REM patients effectively worsened the AngII-mediated cardiomyocyte enlargement. Conversely, we did not observe similar effect after treatment with T1 pEXOs isolated from HiR-REM. As shown in Supplementary Figure 3, the anti-hypertrophic effect of postoperative HiR-REM plasma exosomes was confirmed by normalization of mRNA levels of ANP, BNP, cardiac β-MHC (MYH7), and SERCA2a during AngII exposure. Consistent with the morphological data, a similar dose of postoperative LoR-REM pEXOs did not counteract the AngII-induced increase in the expression of the above genes.

**FIGURE 7:**
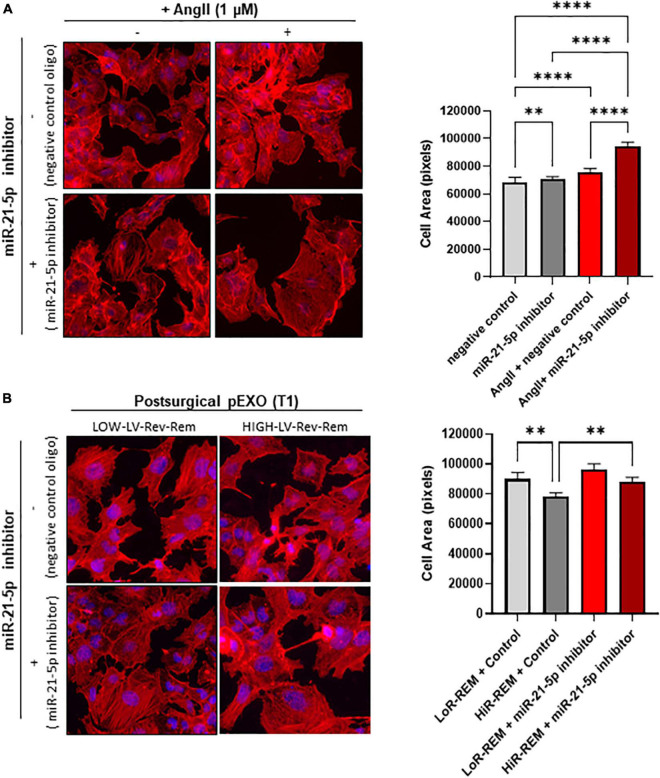
Effect of miR-21-5p inhibition on cardiomyocytes size. The HL-1 cells were seeded at a concentration of 5,000 cells/cm^2^ and allowed to adhere for 24 h before treatment. **(A)** The HL-1 cells were treated with ± 1-μM angiotensin II (AngII, Sigma) in complete antibody-free Claycomb medium for 48 h. Moreover, 24 h after the beginning of the treatment, 30-nM miRNA-21-5p inhibitor/negative control was added to the cells using Lipofectamine^®^ RNAiMAX Transfection Reagent and transfection was maintained for the following 24 h. **(B)** To stimulate hypertrophy, HL-1 cells were treated with 1-μM angiotensin II (AngII, Sigma) in complete antibody-free Claycomb medium for 48 h. The pEXOs isolated at T1 from LoR-REM or HiR-REM patients were loaded with miRNA-21-5p inhibitor/negative control through a heat shock-mediated protocol, as described in the experimental procedure, then collected by ultracentrifugation. Isolated pEXOs were added at a final concentration of 1 × 10^9^ particles/ml 24 h after the beginning of the AngII treatment, and treatment by pEXOs was maintained for the remaining 24 h in the presence of AngII. **(A,B)** At the end of each treatment, the cell monolayer was stained with Phalloidin–Atto 550 as described in the methods. The images were acquired with a fluorescence microscope (Leica; 20× magnification); the cell areas were manually measured with ImageJ software (https://imagej.nih.gov/ij/) and expressed in pixels. Statistical significance of the differences between treatments was evaluated by one-way ANOVA. ^**^p < 0.01; ^****^p < 0.0001.

Because miR-21-5p levels are 10-fold higher than those of other exosomal miRNAs and peak only in pEXOs isolated from HiR-REM patients, we assessed miR-21-5p role during cardiomyocyte response to chronic AngII stimulation. For this purpose, we have performed additional experiments by co-treating cardiomyocytes with miR-21-5p inhibitor. As showed in Figure 7A, miR-21-5p inhibitor promotes a slight enlargement of resting cardiomyocytes and further enhances the AngII-mediated hypertrophic response of note, miR-21-5p inhibitor counteracted the anti-hypertrophic effect of T1-pEXOs isolated from HiR-REM in AngII-treated HL1 cadiomyocytes (Figure 7B).

## Discussion

In the last decade, plasma exosomes have gained increasing interest as perioperative diagnostic, therapeutic and prognostic factors related to different types of cardiac surgery (38, 39). Yet, their role in the cardiac remodeling during the late postoperative period is unknown and attracts attention. Our study demonstrated that the extent of LV Rev–Rem at 6 months after timely and technically successful MVR surgery is related to a different profile of circulating exosomes.

Importantly, we observed an elevated pEXOs values only in patients with a higher grade of reverse remodeling of the left ventricle characterized by lower values of LVEDVi, LVESVi, and ventricular mass index. Since the release of exosomes can be induced by external factors, we can assume that pre-existing diseases and perioperative factors did not affect late exosomal levels after heart valve surgery. All surgical patients with severe primary MR showed similar preoperative LVEF values and were treated with the same drugs and anesthetics. Indeed, preoperative pEXOs level did not change among patients and were independent of the magnitude of regurgitant volume. Of note, preoperative regurgitation volume was higher in patients who exhibited the greatest postoperative LV Rev–Rem. It is conceivable that with the severity of mitral valve disease, the extent of preoperative regurgitant volume associated with adverse remodeling, co-morbidities, as well as co-medications, did not influence the pEXOs level before surgery. We cannot exclude that late release of exosomes was part of a stress response following exposure to additional, as yet undefined, intra- and postoperative factors. In our previous study, indeed, we ruled out that anesthesia and minimally invasive on-pump mitral valve surgery lead to an early increase in postoperative pEXOs level in patients without ischemic heart disease (39). Yet, the causal relationship between circulating exosomes and late adverse or reverse ventricular remodeling after surgery is not yet clarified. Understanding the interplay between plasma exosomes levels and different magnitudes of late reverse cardiac remodeling in patients with entirely competent mitral valve after early surgical repair is essential to develop better-tailored interventions of cardioprotection.

Our study suggests that rising postoperative pEXOs level is a reliable indicator of the long-term adaptive response of the heart after mitral valve repair regardless of LV ejection fraction. Indeed, at 6 months after early rescue surgery, among patients with null mitral regurgitation volumes and preserved systolic function, the HiR-REM group showed smaller ventricular volumes and cardiac mass index than the LoR-REM patients. In light of the finding of partial restoration of the left ventricular structure despite full recovery of mitral valve function, we suggest that low postoperative pEXOs level may hinder the maintenance of normal ventricular chamber geometry and mass over time in LoR-REM patients. This finding is clinically relevant because our study population with severe primary chronic MR had adverse tissue remodeling before surgery, as evidenced by the presence of myocardial fibrosis assessed using CMRI. In fact, adverse remodeling is ongoing even if the ventricle is normocontractile and patients are asymptomatic as demonstrated by the presence of myocardial fibrosis (78).

Even though the *in vivo* kinetics of heterogeneous circulating exosomes is still difficult to investigate, the previous experimental evidence has also ascertained that not all cells release highly cardioprotective exosomes. In this regard, we have demonstrated that higher dose of exosomes released from cardiac progenitor cells provides sustained anti-remodeling effects *in vivo* (44, 79). Although further investigation is needed to define the cellular source and kinetics of pEXOs in our patients, this study is supported by a recent report demonstrating that plasma levels of extracellular vesicles are reduced in patients with more adverse cardiac remodeling (80). However, the mechanisms underlying exosome-based late reverse remodeling remain hitherto unknown.

Exosomes have been proposed as vehicle for miRNA-based intercellular communication (40). To demonstrate how targeting the heart with circulating exosomes may contribute to reverse ventricular remodeling during late postoperative period, we measured exosomal levels of miR-1, –21-5p, and –133a, that were relevant in cardiac remodeling and protection (50–53). First, we compared pre- and postoperative exosomal miRNA levels of our patients to those measured in a cohort of eight healthy volunteers. Although exosomal miR-21-5p copy numbers were ten-fold higher than miR-1 and miR-133a values, miR-21-5p was most abundant in postoperative exosomes of all surgical patients compared to control group (Figure 3F). Since exosomes have a very limited capacity to sample the diverse RNA cargo, our findings contribute to better understand the dynamic of exosome-mediated miRNA communication to chronically maintain normal cardiac phenotype after early mitral valve surgery. Because most plasma changes in exosomal miR-21-5p levels are known to occur far from the onset of cardiac injury (81), their longitudinal monitoring may be useful to predict the late favorable LV remodeling as detected by CMRI. Indeed, exosomal miR-21-5p levels in HiR-REM group are higher than in LoR-REM patients and control subjects. Conversely, postoperative exosomal miR-1 and –133a copy numbers in HiR-REM patients were similar to healthy subjects and slightly higher than those of LoR-REM group.

Even if the ROC curve analysis provided evidence about how the T1 values of pEXOs, miR-1, miR-133a and miR-21-5p fairly discriminates between patients with higher or lower reverse ventricular remodeling during late postoperative period (Figure 5), only patients with increased levels of T1-pEXOs showed reduced left ventricular mass indexed for body surface area compared with preoperative values (Supplementary Figure 2). Our finding is clinically relevant because incomplete regression of the LV mass alone is a hallmark of residual adverse remodeling and limits the ability to recover normal LV systolic function over time after mitral valve repair (26). The causal relationships between incomplete reverse left ventricular remodeling and T1-pEXOs levels, however, need to be further clarified. On this merit, we performed additional experiments *in vitro*. First, we found that T1-pEXOs isolated from LoR-REM patients contribute to further enlarge cardiomyocyte size in response to long-term exposure to AngII, but not T1-pEXOs from HiR-REM group. In line with morphological findings, we found that mRNA levels of ANP, BNP, and cardiac β-MHC (MYH7) established indicators of cardiac hypertrophy (82), do not increase in cardiomyocytes long-term treated with postoperative HiR-REM plasma exosomes during exposure to AngII. In contrast, similar doses of postoperative LoR-REM pEXOs did not inhibit the Ang II-induced fetal hypertrophic gene expression program (83). Of note, we focused on mRNA levels of β-MHC since it is well known that AngII induces both hypertrophy of cultured cardiomyocytes and upregulation of β-MHC levels, without marked effects on α-MHC (84).

Second, we evaluated whether exosomal miR-21-5p, which is highly expressed in T1-pEXOs from HiR-REM patients, plays a key role in counteracting the enhancement of adverse cardiomyocyte remodeling. Our hypothesis was supported by the previous studies showing that downregulation of miR-21-5p reduces the protective effects of extracellular vesicles in limiting cardiomyocyte apoptosis (85) and in preventing cardiac dysfunction (86). However, its direct effect on cardiomyocytes size is still lacking. To this end, our *in vitro* experiments demonstrated that treatment of stressed cardiomyocytes with T1-pEXOs of HiR-REM group in the presence of miR-21-5p inhibitor exacerbated the AngII-induced enlargement of cardiomyocyte size. It is noteworthy that miR-21-5p inhibitor alone was able to worsen the size enlargement of nonexosome-treated cardiomyocytes chronically exposed to AngII. Our results suggest that T1-pEXOs enhanced the autoregulatory feedback mediated by endogenous miR-21-5p to limit AngII-induced remodeling of cardiomyocytes. To further corroborate our experimental findings on exosomal miR-21-5p anti-hypertrophic effects, we investigated changes of SERCA2a gene expression in AngII-stressed cardiomyocytes after treatment with postoperative HiR-REM exosomes, which are rich in miR-21-5p. Indeed, it is well known that SERCA2a expression undergoes significant downregulation after AngII-induced hypertrophy (83), and therefore a reduction in its activity, leading to slower cytosolic Ca^2+^ removal, reduced sarcoplasmic reticulum content and impaired Ca^2+^ transient reduction (87). Moreover, it is already known that exosomal miR-21-5p may increase cardiomyocyte-specific SERCA2a expression in both human engineered cardiac tissue and human pluripotent stem cell-derived cardiomyocyte monolayer, while its mRNA levels are decreased by delivery of exosome-enriched fraction from miR-21-5p inhibitor treated cells (88). In our study, the effect detected after long-term exposure of HL-1 to AngII was a modest yet statistically significant reduction of SERCA2a transcription with reference to β-actin transcription. Of note, the transcript level of β-actin was found to be unchanged after chronic exposure to AngII. Treating stressed cardiomyocytes with postoperative HiR-REM exosomes significantly increased SERCA2a at normal levels in comparison to T1-pEXOs isolated from LoR-REM patients. Our first results support miR-21-5p-rich plasma exosomes exerting anti-hypertrophic effects by regulating expression of calcium handling genes in accord with the previous study (88).

In conclusion, our data demonstrated that sustained increase in circulating pEXOs levels in the late postoperative period depicts patients with greater extent of reverse ventricular remodeling after MVR since they deliver highest concentration of miR-21-5p, which may play a key role in counteracting worsening of long-term adverse cardiac remodeling by normalizing SERCA2a gene expression in cardiomyocytes. The late postoperative monitoring of miR-21-5p-rich pEXOs level will improve our understanding of long-term adaptive autoregulatory feedback of cardiomyocytes leading to postoperative reverse remodeling. Finally, our findings will be helpful to design a new approach to early predict and treat patients at higher risk of partial reverse remodeling after a successful surgery. Indeed, miR-21-5p-rich exosomes have emerged from our study as a new precision theranostic tool for late postoperative cardioprotection.

## Limitations of the study

Some limitations of this study should be considered to promote next investigations. First, the study population consists of a low number of patients. This was in part due to the relatively low number of patients with isolated primary MR who underwent surgery within the timeframe of the study and partly due to the low patient adherence to the experimental protocol, mainly driven by the refusal of the CMRI examination due to claustrophobia. However, the high accuracy and reproducibility of CMRI can partly compensate for the small sample size. In fact, each patient studied with CMRI is equivalent to 10 patients studied with echocardiography, whose measurements are less reliable. Second, our population consists mainly of surgical patients in the early stage of adverse cardiac remodeling induced by primary MR, with mild-to-moderate degrees of LV dilatation. In accord with latest recommendations for acting in elective surgical MVR (36), we cannot ignore that it is difficult to enroll patients with higher degrees of adverse ventricular remodeling. However, the mean preoperative CMRI-derived LVEDVi was 113.67 ± 25 ml/m^2^ in our study population, and considering that the upper limit is 95–93 ml/m^2^ in middle-aged male subjects, we can affirm that most of our patients had not severe adverse LV remodeling (15/19 subjects showed LVEDVi above the upper limit). Third, our follow-up was limited to 6 months after surgery. Further investigations are required to assess the relationship between miR-21-5p-rich plasma exosome levels and magnitude of reverse ventricular remodeling during longer follow-up after early rescue MVR. Finally, the definition of the cellular source of miR-21-5p-rich plasma exosomes and better understanding of molecular pathways regulated by exosomal miR-21-5p in cardiomyocytes deserve further *ad hoc* investigation *in vivo*.

## Data availability statement

The original contributions presented in this study are included in the article/Supplementary material, further inquiries can be directed to the corresponding author.

## Ethics statement

The studies involving human participants were reviewed and approved by Ethics Committee of “G. Monasterio” Foundation (FTGM, Massa, Italy; EMIGRATE study, approval n°1529). The patients/participants provided their written informed consent to participate in this study.

## Author contributions

FP, GF, and VL contributed to the conception and design of the study; GF and VC advised in physiological mechanisms. SS, DC, MS, GB, and SM advised in physiological topics and surgical approach. FP and GF performed the statistical analysis. VL analyzed the results and wrote the first draft of the manuscript. GF, GA, FP, MM, and VL wrote sections of the manuscript. All authors contributed to manuscript revision, read, and approved the submitted version.

## Footnotes

1
https://imagej.nih.gov/ij/
