# Supplementary material for: Late plasma exosome microRNA-21-5p depicts magnitude of reverse ventricular remodeling after early surgical repair of primary mitral valve regurgitation
Source: Front Cardiovasc Med. 2022 Jul 29;9:943068. doi: 10.3389/fcvm.2022.943068 (PMC9373041; doi:10.3389/fcvm.2022.943068)
Supplement: Supplementary file 1 [file Data_Sheet_1.pdf]

Supplementary Table 1: Comparison of preoperative and postoperative CMRI results in primary MR patents undergoing MVR surgery. CMRI was performed on N=19 patients undergoing MVR surgery before (T0) and 6 months after (T1) procedure. Data are presented as mean ± SD. Statistical significance of the differences between T0 and T1 was evaluated by paired Student's T test or Wilcoxon test depending on data distribution.

	**T0**	**T1**		**p-value**
**LVEDVi** [mL/m^2]	113.67 ± 25.37	73.52 ± 12.83	▼	**1.90·10^{-6}**
**LVESVi** [mL/m^2]	42.50 ± 11.77	35.51 ± 9.56	▼	**0.0055**
**RVEDVi** [mL/m^2]	84.78 ± 15.57	66.92 ± 10.92	▼	**1.30·10^{-5}**
**RVESVi** [mL/m^2]	34.44 ± 9.00	30.21 ± 7.50	▼	**0.0352**
**LV Mass index** [g/m^2]	77.44 ± 13.36	70.55 ± 10.02	▼	**0.0035**
**LVSV** [mL]	142.33 ± 35.93	74.84 ± 16.74	▼	**7.14·10^{-7}**
**LVSVi** [mL/m^2]	72.36 ± 16.98	38.02 ± 7.64	▼	**7.26·10^{-7}**
**RVSV** [mL]	100.67 ± 20.93	72.32 ± 16.80	▼	**3.10·10^{-5}**
**RVSVi** [mL/m^2]	51.15 ± 9.72	36.71 ± 7.57	▼	**3.70·10^{-5}**
**LVEF** [%]	62.60 ± 5.87	52.01 ± 7.82	▼	**2.80 ·10^{-7}**
**RVEF** [%]	59.64 ± 6.56	54.89 ± 7.76	▼	**0.0215**
**Heart rate** [BPM]	61.00 ± 7.92	70.47 ± 12.89	▲	**0.0100**
**Cardiac index** [mL/min ·m^2]	4180.36 ± 1114.27	2638.26 ± 555.51	▼	**7.40·10^{-5}**
**LA Area index** [cm]	19.37 ± 7.89	9.95 ± 2.17	▼	**7.60·10^{-6}**
**RA Area** [cm]	14.32 ± 5.71	10.58 ± 2.32	▼	**0.0010**
**LA-max volume index** [mL/m^2]	77.53 ± 27.15	37.77 ± 7.47	▼	**1.30·10^{-5}**
**LAEF** [%]	53.44 ± 9.48	37.37 ± 10.83	▼	**0.00067**

Supplementary Table 2: Analysis of CMRI parameters in MR patients showing different rates of reverse remodeling after MVR surgery. CMRI data were collected for N=19 patients undergoing MVR surgery before (T0) and 6 months after (T1) the procedure. For each CMRI parameter measured, percentage change between T0 and T1 was computed as %Δ = (T1–T0) / T0 * 100 for each patient. T0, T1 and percentage change CMRI data (%Δ) were compared between LoR-REM and HiR-REM patients. Data are presented as mean ± SD. Statistical significance of the differences was evaluated by independent samples Student's T test (with or without Welch's correction) or Mann Whitney test depending on data distribution.

		**LoR-REV**	**HiR-REV**		**p-value**
**LVEDVi**	T0 [mL/m²]	100.05 ± 13.34	125.93 ± 27.87	▲	0.0209
	T1 [mL/m²]	79.11 ± 8.67	68.50 ± 14.27	Ns	0.1774
	**Δ (T1-T0)/ T0 [%]**	**-20.23 ± 9.86**	**-44.61 ± 9.94**	▼	**$5.20 \cdot 10^{-5}$**
**LVESVi**	T0 [mL/m²]	39.82 ± 11.69	44.92 ± 11.92	Ns	0.3582
	T1 [mL/m²]	39.67 ± 9.45	31.90 ± 8.46	Ns	0.0758
	**Δ (T1-T0)/ T0 [%]**	**2.40 ± 21.87**	**-28.40 ± 10.97**	▼	**0.0010**
**RVEDVi**	T0 [mL/m²]	83.97 ± 13.40	85.52 ± 18.01	Ns	0.8340
	T1 [mL/m²]	70.00 ± 8.66	64.10 ± 12.35	Ns	0.2496
	**Δ (T1-T0)/ T0 [%]**	**-15.43 ± 12.70**	**-23.82 ± 12.44**	**Ns**	**0.1642**
**RVESVi**	T0 [mL/m²]	34.67 ± 9.29	34.24 ± 9.24	Ns	0.9213
	T1 [mL/m²]	31.67 ± 8.03	28.90 ± 7.16	Ns	0.3234
	**Δ (T1-T0)/ T0 [%]**	**-5.419 ± 25.49**	**-12.10 ± 22.84**	**Ns**	**0.5546**
**LV Mass index**	T0 [g/m²]	74.56 ± 7.57	80.04 ± 17.04	Ns	0.3727
	T1 [g/m²]	72.94 ± 6.99	68.40 ± 12.09	Ns	0.3373
	**Δ (T1-T0)/ T0 [%]**	**-1.928 ± 6.47**	**-13.43 ± 9.511**	▼	**0.0073**
**LVSVi**	T0 [mL/m²]	60.26 ± 6.32	84.46 ± 15.60	▲	0.0013
	T1 [mL/m²]	39.56 ± 7.21	36.60 ± 8.11	Ns	0.3030
	**Δ (T1-T0)/ T0 [%]**	**-34.53 ± 9.70**	**-52.95 ± 12.70**	▼	**0.0032**
**RVSVi**	T0 [mL/m²]	49.33 ± 7.11	52.98 ± 11.94	Ns	0.4423
	T1 [mL/m²]	38.33 ± 5.70	35.30 ± 8.98	Ns	0.3981
	**Δ (T1-T0)/ T0 [%]**	**-21.24 ± 14.72**	**-28.37 ± 14.31**	**Ns**	**0.3133**
**LVEF**	T0 [%]	60.73 ± 7.21	64.28 ± 4.01	Ns	0.2114
	T1 [%]	50.33 ± 9.14	53.50 ± 6.29	Ns	0.3870
	**Δ (T1-T0)/ T0 [%]**	**-17.64 ± 9.94**	**-16.50 ± 9.64**	**Ns**	**0.8030**
**RVEF**	T0 [%]	59.12 ± 6.37	60.10 ± 7.02	Ns	0.7793
	T1 [%]	54.89 ± 8.42	54.70 ± 7.39	Ns	0.9591
	**Δ (T1-T0)/ T0 [%]**	**-6.420 ± 13.19**	**-8.296 ± 12.63**	**Ns**	**0.7554**
**Heart rate**	T0 [BPM]	62.67 ± 9.26	59.50 ± 6.64	Ns	0.3998
	T1 [BPM]	67.11 ± 14.22	73.50 ± 11.46	Ns	0.2937
	**Δ (T1-T0)/ T0 [%]**	**8.353 ± 23.14**	**24.71 ± 22.66**	**Ns**	**0.1382**
**Cardiac index**	T0 [mL/min ·m²]	3,498.15 ± 703.51	4,794.35 ± 1,076.56	▲	0.0070
	T1 [mL/min ·m²]	2582 ± 397.3	2689 ± 686.2	Ns	0.6874
	**Δ (T1-T0)/ T0 [%]**	**-20.93 ± 33.02**	**-41.55 ± 17.17**	**Ns**	**0.1128**
**LA Area**	T0 [cm]	18.89 ± 7.20	19.80 ± 8.83	Ns	0.9483
	T1 [cm]	10.56 ± 2.55	9.40 ± 1.71	Ns	0.3352
	**Δ (T1-T0)/ T0 [%]**	**-38.80 ± 21.70**	**-47.26 ± 14.97**	**Ns**	**0,3325**
**RA Area**	T0 [cm]	17.11 ± 7.17	11.80 ± 2.15	Ns	0.0602
	T1 [cm]	11.33 ± 2.12	9.90 ± 2.38	Ns	0.1855
	**Δ (T1-T0)/ T0 [%]**	**-25.65 ± 23.03**	**-15.33 ± 17.95**	**Ns**	**0.2885**
**LA-max volume index**	T0 [mL/m²]	62.67 ± 12.33	89.09 ± 30.41	▲	0.0371
	T1 [mL/m²]	38.01 ± 5.03	37.55 ± 9.44	Ns	0.8981
	**Δ (T1-T0)/ T0 [%]**	**-34.97 ± 15.45**	**-53.12 ± 11.58**	▼	**0.0175**
**LAEF**	T0 [%]	52.29 ± 6.07	54.33 ± 11.78	Ns	0.8980
	T1 [%]	37.44 ± 10.47	37.30 ± 11.71	Ns	0.9778
	**Δ (T1-T0)/ T0 [%]**	**-27.03 ± 20.15**	**-28.07 ± 24.50**	**Ns**	**0.9291**

Supplementary Table 3: Analysis of pEXO and exosomal miRNA in MR patients showing different rates of reverse remodeling after MVR surgery. pEXO were isolated from MR patients' (N=19) plasma collected before (T0) and 6 months after surgery (T1); N=8 healthy controls were also employed. pEXO were quantified by NTA as described in the methods. Exosomal miRNA-1, miRNA-21 and miRNA-133a were amplified and measured by qRT-PCR as described in the methods. Presurgical (T0) and postsurgical (T1) levels, and percentage perioperative change [(T1-T0)/T0 *100] were compared between LoR-REM and HiR-REM patients. Data are presented as median with interquartile range. Statistical significance of the differences between patients' groups was evaluated by independent samples Student's T test (with or without Welch's correction) or Mann Whitney test depending on data distribution. Statistical differences between patients and controls was evaluated by one-way ANOVA (with or without Brown-Forsythe modification for heteroscedastic groups) or Kruskal-Wallis test, depending on sample distribution; p'= p-value LOW-Rev-Rem vs Controls; p''=p-value HIGH-Rev-rem vs Controls.

		**LoR-REV**	**HiR-REV**	**p-value**	**Controls**	**p'**	**p''**
**pEXO**	**T0 [particles/mL]**	$1.36\cdot10^9$ ($7.99\cdot10^8$ - $1.99\cdot10^9$)	$1.48\cdot10^9$ ($6.92\cdot10^8$ - $2.89\cdot10^9$)	0.6607	$1.08\cdot10^9$ ($8.78\cdot10^8$ - $1.39\cdot10^9$)	0.8522	0.5013
	**T1 [particles/mL]**	$1.31\cdot10^9$ ($7.52\cdot10^8$ - $1.57\cdot10^9$)	$1.89\cdot10^9$ ($1.43\cdot10^8$ - $2.49\cdot10^9$)	**0.0625**	$1.08\cdot10^9$ ($8.78\cdot10^8$ - $1.39\cdot10^9$)	>0.9999	**0.0159**
	**Δ (T1-T0)/ T0 [%]**	-4.62 (-56.76 - 137.24)	26.03 (-12.46 - 206.38)	0.4470			
**miR-1**	**T0 [$2^{-\Delta\Delta CT}$]**	1.137 (0.339 - 1.838)	1.405 (1.058 - 2.732)	0.2428	0.771 (0.312 - 2.431)	>0.9999	0.2685
	**T1 [$2^{-\Delta\Delta CT}$]**	0.313 (0.0034 – 1.105)	1.184 (0.594 - 2.386)	**0.0179**	0.771 (0.312 - 2.431)	0.1795	0.8747
	**Δ (T1-T0)/ T0 [%]**	-75.52 (-99.17 - 13.52)	-27.78 (-66.94 - 100.73)	0.1487			
**miR-21**	**T0 [$2^{-\Delta\Delta CT}$]**	1.993 (0.946 - 6.285)	2.485 (1.274 - 8.530)	0.7209	1.053 (0.419 - 2.081)	0.4062	0.5777
	**T1 [$2^{-\Delta\Delta CT}$]**	2.289 (0.777 – 6.101)	9.314 (1.512 - 13.081)	**0.0303**	1.053 (0.419 - 2.081)	0.4749	**0.0251**
	**Δ (T1-T0)/ T0 [%]**	14.87 (-32.90 - 52.75)	56.49 (-40.64 - 386.57)	0.2973			
**miR-133a**	**T0 [$2^{-\Delta\Delta CT}$]**	0.772 (0.364 - 2.110)	0.962 (0.521 - 1.925)	0.3562	1.205 (0.418 - 2.536)	0.7094	>0.9999
	**T1 [$2^{-\Delta\Delta CT}$]**	0.456 (0.3657 – 1.081)	1.835 (0.5974 - 3.964)	**0.0464**	1.205 (0.418 - 2.536)	0.2882	>0.9999
	**Δ (T1-T0)/ T0 [%]**	-13.31 (-55.74 - 46.80)	11.26 (-36.49 - 179.10)	0.2107			

Supplementary Table 4: **Analysis of CMRI parameters in MR patients with upregulated or downregulated postoperative levels of pEXO.** CMRI and pEXO data were collected from MR patients (N=19) before (T0) and 6 months after MVR surgery (T1); isolated pEXO were quantified by NTA as described in the methods. Percentage of change in pEXO was calculated as %Δ = (T1-T0)/T0 * 100, and patients were grouped depending on exosome decrease (DOWN, %Δ <0) or increase (UP, %Δ >0) after surgery. Percentage variation of CMRI parameters between the presurgical scan (T0) and the postsurgical follow up (T1) was calculated as %Δ =(T1-T0)/T0 * 100. T0, T1 and percentage change CMRI data (%Δ) were compared between patients with exosome downregulation (DOWN) and patients with exosome increase (UP) after surgery. Data are presented as mean ± SD. Statistical significance of the differences was evaluated by independent samples Student's T test (with or without Welch's correction) or Mann Whitney test depending on data distribution.

		**Decrease in exosome secretion (DOWN)**	**Increase in exosome secretion (UP)**		**p-value**
**LVEDVi**	**T0** [mL/m^2]	116.7 ± 27.07	111.9 ± 25.38	Ns	0.6995
	T1 [mL/m^2]	79.57 ± 6.60	70.00 ± 14.46	Ns	0.1195
	**Δ (T1-T0)/ T0 [%]**	**-30.02 ± 9.42**	**-34.84 ± 18.71**	**Ns**	**0.5367**
**LVESVi**	T0 [mL/m^2]	46.43 ± 14.98	40.21 ± 9.45	Ns	0.2794
	T1 [mL/m^2]	39.57 ± 8.32	33.25 ± 9.78	Ns	0.1707
	**Δ (T1-T0)/ T0 [%]**	**-11.91 ± 13.56**	**-14.92 ± 27.41**	**Ns**	**0.5358**
**RVEDVi**	T0 [mL/m^2]	85.91 ± 13.50	84.12 ± 17.20	Ns	0.8160
	T1 [mL/m^2]	69.00 ± 7.85	65.67 ± 12.50	Ns	0.5356
	**Δ (T1-T0)/ T0 [%]**	**-18.38 ± 12.46**	**-20.70 ± 13.69**	**Ns**	**0.7171**
**RVESVi**	T0 [mL/m^2]	34.89 ± 7.935	34.18 ± 9.90	Ns	0.8751
	T1 [mL/m^2]	28.43 ± 7.57	31.25 ± 7.59	Ns	0.4449
	**Δ (T1-T0)/ T0 [%]**	**-16.34 ± 20.68**	**-4.62 ± 25.10**	**Ns**	**0.3116**
**LV Mass index**	T0 [g/m^2]	78.10 ± 14.00	77.05 ± 13.59	Ns	0.8742
	T1 [g/m^2]	76.16 ± 10.20	67.28 ± 8.69	Ns	0.0598
	**Δ (T1-T0)/ T0 [%]**	**-1.75 ± 7.10**	**-11.62 ± 9.74**	▼	**0.0323**
**LVSVi**	T0 [mL/m^2]	70.29 ± 16.49	73.73 ± 17.99	Ns	0.6886
	T1 [mL/m^2]	40.14 ± 8.82	36.75 ± 6.96	Ns	0.4030
	**Δ (T1-T0)/ T0 [%]**	**-41.93 ± 12.58**	**-44.90 ± 16.08**	**Ns**	**0.6856**
**RVSVi**	T0 [mL/m^2]	51.00 ± 8.00	51.18 ± 11.16	Ns	0.9707
	T1 [mL/m^2]	40.57 ± 5.25	34.50 ± 7.98	Ns	0.0916
	**Δ (T1-T0)/ T0 [%]**	**-19.93 ± 9.91**	**-27.90 ± 16.55**	**Ns**	**0.2698**
**LVEF**	T0 [%]	60.57 ± 7.57	63.75 ± 4.53	Ns	0.2644
	T1 [%]	50.57 ± 10.29	52.83 ± 6.13	Ns	0.5530
	**Δ (T1-T0)/ T0 [%]**	**-17.11 ± 11.57**	**-16.99 ± 8.69**	**Ns**	**0.9806**
**RVEF**	T0 [%]	59.71 ± 5.85	59.50 ± 7.28	Ns	0.9480
	T1 [%]	58.86 ± 7.71	52.42 ± 6.87	Ns	0.0764
	**Δ (T1-T0)/ T0 [%]**	**-1.03 ± 8.88**	**-11.13 ± 13.21**	**Ns**	**0.0911**
**Heart rate**	T0 [BPM]	60.00 ± 6.63	61.58 ± 8.82	Ns	0.6867
	T1 [BPM]	66.86 ± 15.51	72.58 ± 11.30	Ns	0.3653
	**Δ (T1-T0)/ T0 [%]**	**10.83 ± 17.73**	**20.54 ± 26.75**	**Ns**	**0.4062**
**Cardiac index**	T0 [mL/min ·m^2]	4176 ± 791.3	4183 ± 1300	Ns	0.9907
	T1 [mL/min ·m^2]	2581 ± 330.0	2671 ± 665.0	Ns	0.7435
	**Δ (T1-T0)/ T0 [%]**	**-37.29 ± 7.73**	**-28.57 ± 33.97**	**Ns**	**0.4097**
**LA Area**	T0 [cm]	19.29 ± 7.16	19.42 ± 8.60	Ns	0.8857
	T1 [cm]	9.714 ± 1.70	10.08 ± 2.47	Ns	0.9056
	**Δ (T1-T0)/ T0 [%]**	**-45.04 ± 18.51**	**-42.21 ± 19.15**	**Ns**	**0.7565**
**RA Area**	T0 [cm]	16.29 ± 7.87	13.17 ± 3.95	Ns	0.6939
	T1 [cm]	11.14 ± 2.48	10.25 ± 2.26	Ns	0.4335
	**Δ (T1-T0)/ T0 [%]**	**-23.22 ± 20.78**	**-18.47 ± 21.23**	**Ns**	**0.6413**
**LA-max volume index**	T0 [mL/m^2]	74.18 ± 13.39	79.55 ± 33.42	Ns	0.7156
	T1 [mL/m^2]	40.22 ± 6.51	36.34 ± 7.88	Ns	0.2867
	**Δ (T1-T0)/ T0 [%]**	**-43.03 ± 8.86**	**-46.48 ± 19.34**	**Ns**	**0.6895**
**LAEF**	T0 [%]	48.83 ± 3.54	56.20 ± 10.96	Ns	0.0740
	T1 [%]	34.86 ± 10.29	38.83 ± 11.30	Ns	0.4559
	**Δ (T1-T0)/ T0 [%]**	**-24.50 ± 18.48**	**-29.48 ± 24.59**	**Ns**	**0.6765**

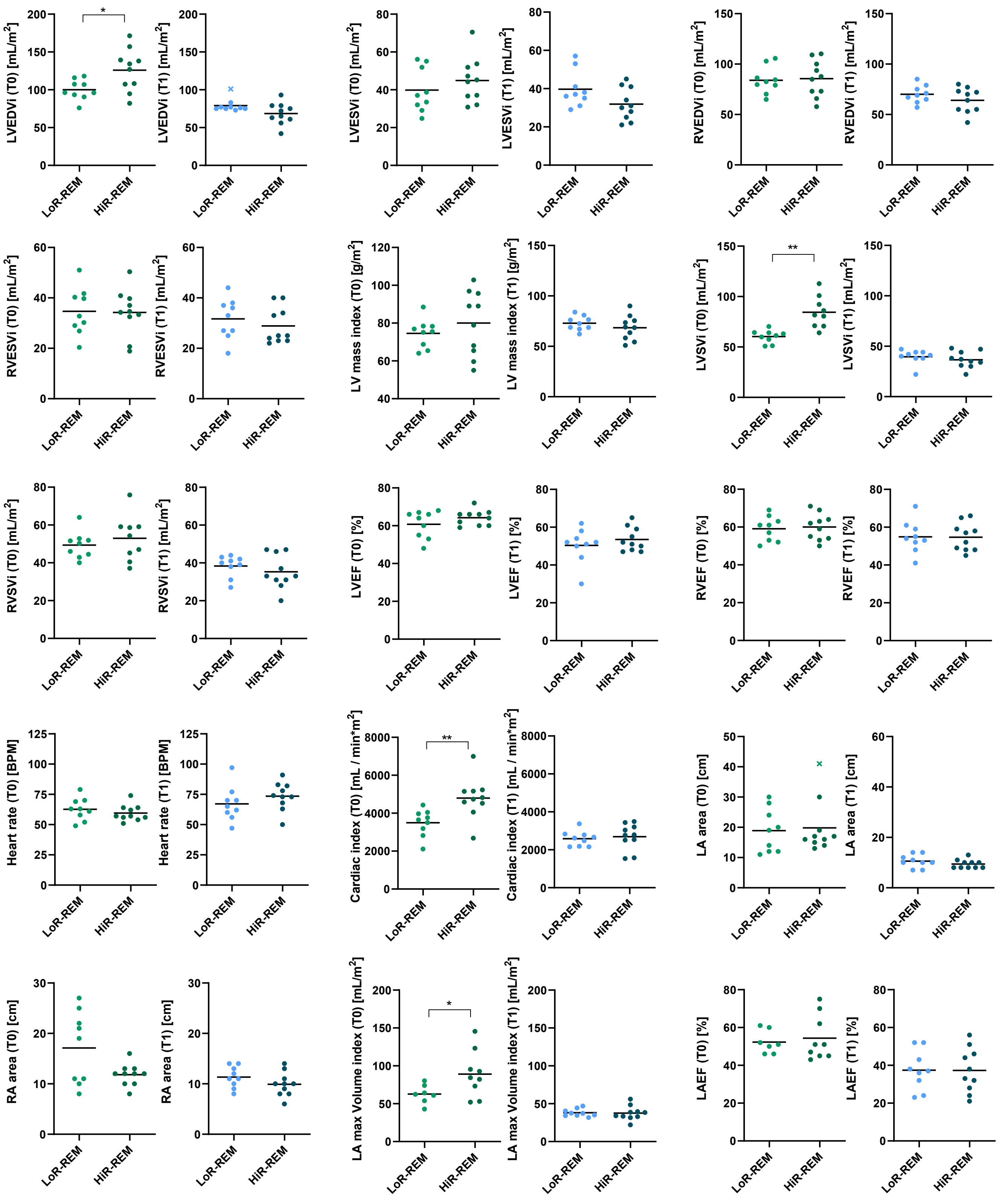

Supplementary Figure 1: Analysis of preoperative (T0) and postoperative (T1) CMRI parameters in MR patients showing different rates of reverse remodeling after MVR surgery. CMRI data were collected for N=19 patients undergoing MVR surgery before (T0) and 6 months after (T1) the procedure. Data were compared between LoR-REM and HiR-REM patients. Individual data are shown, horizontal bars represent group average. Statistical significance of the differences was evaluated by independent samples Student's T test (with or without Welch's correction) or Mann Whitney test depending on data distribution. *, $p<0.05$; **, $p<0.01$; ***, $p<0.001$; ****, $p<0.0001$.

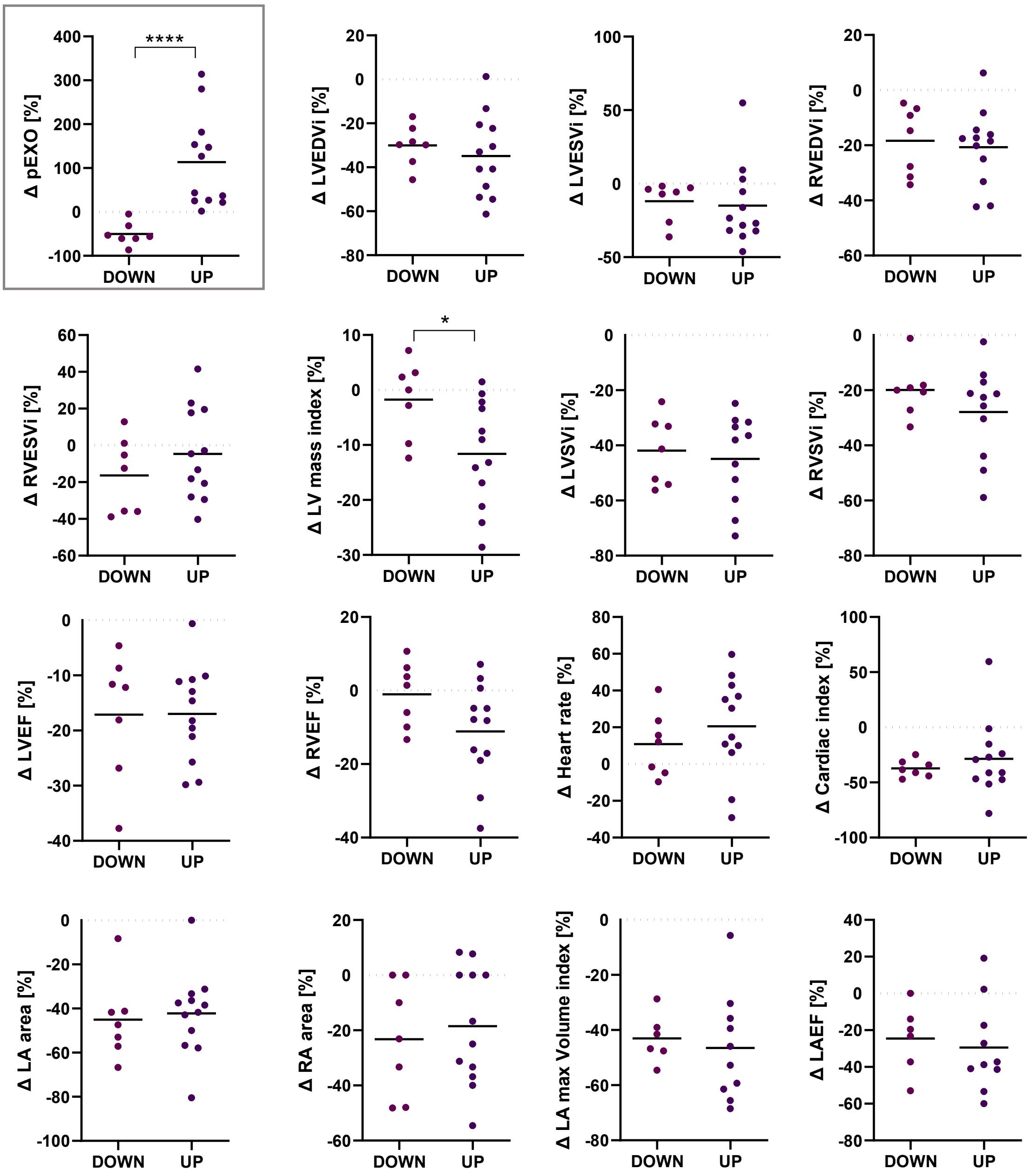

Supplementary Figure 2: Analysis of CMRI parameters in MR patients with upregulated or downregulated postoperative levels of pEXO. CMRI and pEXO data were collected from MR patients (N=19) before (T0) and 6 months after MVR surgery (T1); isolated pEXO were quantified by NTA as described in the methods. Percentage of change in pEXO was calculated as %Δ = (T1-T0)/T0 * 100, and patients were grouped depending on exosome decrease (DOWN, %Δ <0) or increase (UP, %Δ >0) after surgery. Percentage variation of CMRI parameters between the presurgical scan (T0) and the postsurgical follow up (T1) was calculated as %Δ =(T1-T0)/T0 * 100. Data are compared between patients with exosome downregulation (DOWN) and patients with exosome increase (UP) after surgery. Individual data are shown; horizontal bars represent group average. Statistical significance of the differences was evaluated by independent samples Student's T test (with or without Welch's correction) or Mann Whitney test depending on data distribution. *, $p<0.05$; **, $p<0.01$; ***, $p<0.001$; ****, $p<0.0001$.

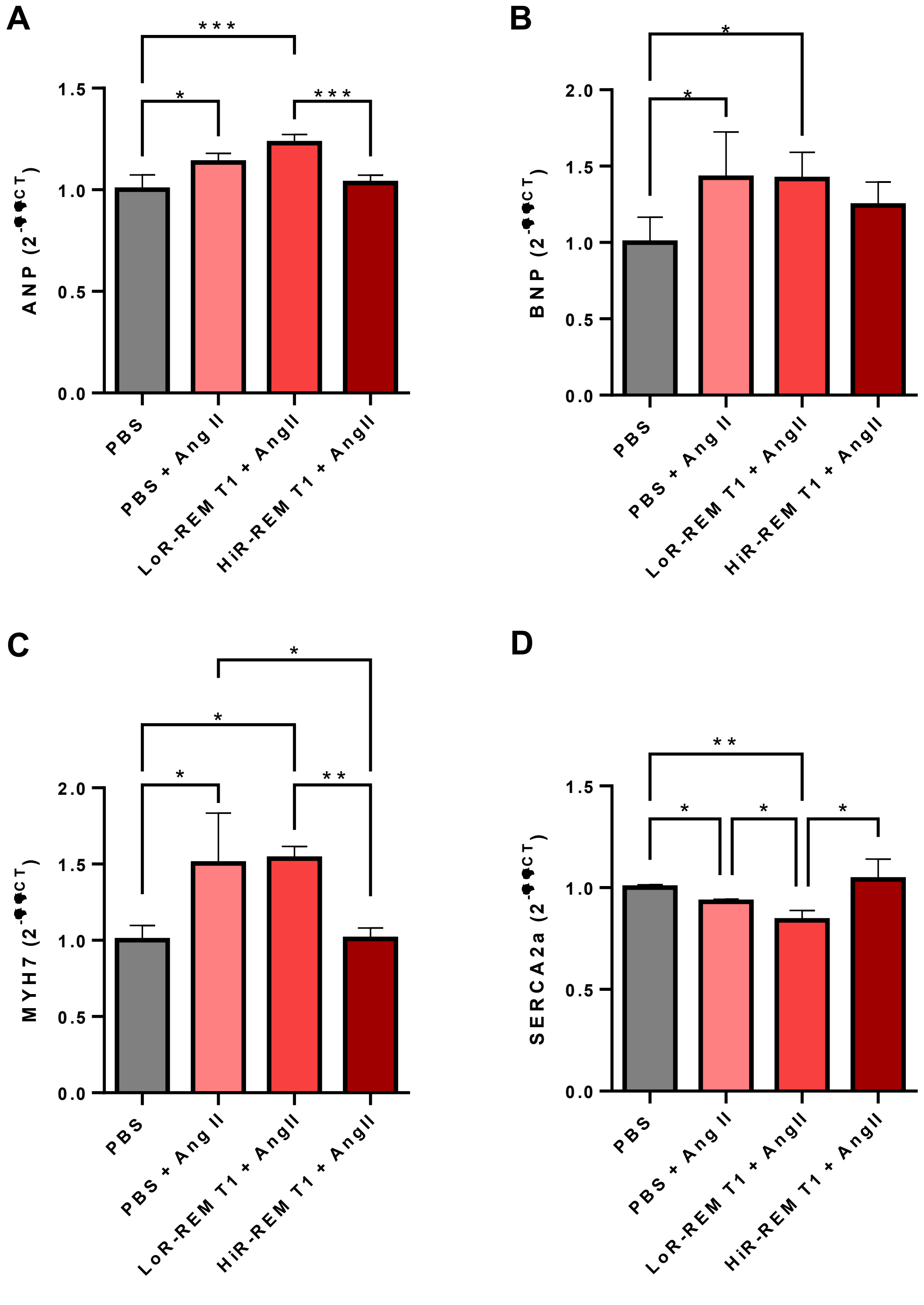

Supplementary figure 3: Effect of pEXOs from patients with different grade of reverse ventricular remodeling on cardiomyocytes size. HL-1 cells were seeded at a concentration of 5,000 cells / cm^2 and allowed to adhere for 24 h before treatment. Cells were treated with ± 1 µM Angiotensin II (AngII, Sigma) in complete Claycomb medium for 48 hours. A PBS suspension of pEXOs isolated from HiR- or LoR-REM group at T1 was added to a final concentration of $1 \cdot 10^9$ particles / mL, 24 h after the beginning of the treatment, and the treatment was maintained for the remaining 24 h; PBS was employed as a control. RNA was isolated using using PRImeZOL™ reagent and RT-PCR performed as described in the methods. Relative expression of specific markers is presented in the figure: A) ANP; B)BNP; C) β-MHC (MYH7) D) SERCA2a.
